# Local and global Cdc42 guanine nucleotide exchange factors for fission yeast cell polarity are coordinated by microtubules and the Tea1–Tea4–Pom1 axis

**DOI:** 10.1242/jcs.216580

**Published:** 2018-07-19

**Authors:** Ye Dee Tay, Marcin Leda, Andrew B. Goryachev, Kenneth E. Sawin

**Affiliations:** 1 Wellcome Centre for Cell Biology, School of Biological Sciences, University of Edinburgh, Michael Swann Building, Max Born Crescent, Edinburgh EH9 3BF, UK; 2 SynthSys – Centre for Synthetic and Systems Biology, School of Biological Sciences, University of Edinburgh, CH Waddington Building, Max Born Crescent, Edinburgh EH9 3BF, UK

**Keywords:** Cdc42, Cell polarity, Fission yeast, Guanine nucleotide exchange factor, Microtubules

## Abstract

The conserved Rho-family GTPase Cdc42 plays a central role in eukaryotic cell polarity. The rod-shaped fission yeast *Schizosaccharomyces pombe* has two Cdc42 guanine nucleotide exchange factors (GEFs), Scd1 and Gef1, but little is known about how they are coordinated in polarized growth. Although the microtubule cytoskeleton is normally not required for polarity maintenance in fission yeast, we show here that when *scd1* function is compromised, disruption of microtubules or the polarity landmark proteins Tea1, Tea4 or Pom1 leads to disruption of polarized growth. Instead, cells adopt an isotropic-like pattern of growth, which we term PORTLI growth. Surprisingly, PORTLI growth is caused by spatially inappropriate activity of Gef1. Although most Cdc42 GEFs are membrane associated, we find that Gef1 is a broadly distributed cytosolic protein rather than a membrane-associated protein at cell tips like Scd1. Microtubules and the Tea1–Tea4–Pom1 axis counteract inappropriate Gef1 activity by regulating the localization of the Cdc42 GTPase-activating protein Rga4. Our results suggest a new model of fission yeast cell polarity regulation, involving coordination of ‘local’ (Scd1) and ‘global’ (Gef1) Cdc42 GEFs via microtubules and microtubule-dependent polarity landmarks.

## Abstract (precis)

**Highlighted Article:** Cell polarity in fission yeast is regulated by two different Cdc42 guanine nucleotide exchange factors, coordinated by the microtubule-dependent landmark system.

## INTRODUCTION

Cell polarity is essential for many eukaryotic cell functions, including migration and/or directional growth, intracellular transport, cell signaling, asymmetric cell division and tissue organization (Campanale et al., 2017; Mayor and Etienne-Manneville, 2016; Rodriguez-Boulan and Macara, 2014; Schelski and Bradke, 2017; St Johnston and Ahringer, 2010). Cell polarization involves generation of spatial cues (intrinsic or extrinsic) for polarity site selection, recruitment of specific proteins to regions of plasma membrane, and reorganization of the actin and microtubule cytoskeleton and of intracellular trafficking. The Rho-family GTPase Cdc42 has important roles in many of these processes (Chiou et al., 2017; Etienne-Manneville, 2004, 2013; Hall, 2012; Harris and Tepass, 2010; Martin and Arkowitz, 2014; Perez and Rincón, 2010). Like other small GTPases, Cdc42 binds effector proteins in its active, GTP-bound state. Control of Cdc42 activity by GTPase-activating proteins (GAPs) and guanine nucleotide exchange factors (GEFs) is thus a crucial feature of polarity regulation (Bos et al., 2007; Cook et al., 2014; Hodge and Ridley, 2016; Moon and Zheng, 2003; Rossman et al., 2005).

Unicellular eukaryotes, such as budding yeast *Saccharomyces cerevisiae* and fission yeast *Schizosaccharomyces pombe*, are excellent models for studying Cdc42-dependent cell polarity, owing to their simple geometries and reduced complexity relative to metazoans (Chiou et al., 2017; Martin and Arkowitz, 2014). Budding yeast are ovoid and form a single bud once per cell cycle, whereas fission yeast are rod-shaped and grow at their tips. In recent years, work in budding yeast has led to key insights into the mechanism(s) by which a stable, self-organized polarity cluster based on Cdc42-GTP can emerge on the plasma membrane to establish a presumptive bud site (Chiou et al., 2017; Goryachev and Leda, 2017; Woods and Lew, 2017). Cdc42 cluster formation depends on spontaneous symmetry-breaking via multiple converging positive feedback loops involving active Cdc42, Cdc42 effectors and the Cdc42 GEF, Cdc24. Local enrichment of these factors via positive feedback can be sufficient for the establishment of cell polarity at a site designated by internal and/or external cues (Chiou et al., 2017; Goryachev and Leda, 2017; Woods and Lew, 2017).

Although many of the components and mechanisms involved in budding yeast polarity are conserved in fission yeast, there are also distinct differences. Scd1, the fission yeast ortholog of budding yeast Cdc24, is thought to have a similar role to Cdc24, functioning in a positive feedback loop to organize polarity clusters of Cdc42–GTP on the plasma membrane at cell tips (Chang et al., 1999, 1994; Chiou et al., 2017; Endo et al., 2003). However, while Cdc24 is essential for viability, Scd1 is nonessential, as fission yeast has a second Cdc42 GEF, Gef1. Scd1 and Gef1 are thought to share an overlapping essential function, because single-deletion mutants of either gene (*scd1*Δ or *gef1*Δ) are viable, whereas the double-deletion mutant (*scd1*Δ *gef1*Δ) is lethal (Coll et al., 2003; Hirota et al., 2003).

Both Scd1 and Gef1 have been described to localize to the cell midzone during cytokinesis and to the cell tips during interphase (Coll et al., 2003; Das et al., 2009, 2015; Hirota et al., 2003; Kokkoris et al., 2014; Vjestica et al., 2013). However, phenotypes associated with Scd1 and Gef1 differ significantly*.* Unlike rod-shaped wild-type cells, *scd1*Δ cells have a mostly round morphology (Chang et al., 1994) and lack detectable enrichment of Cdc42-GTP at cell tips (Kelly and Nurse, 2011; Tatebe et al., 2008). By contrast, *gef1*Δ cells have a largely wild-type morphology, albeit with mild defects in bipolar tip growth and septum formation (Coll et al., 2003). Similarly, Scd1 overexpression leads to no significant change in cell morphology, whereas Gef1 overexpression causes cells to become wider or rounder (Coll et al., 2003; Das et al., 2012). It is currently unclear how Scd1 and Gef1 activities are coordinated in the activation of Cdc42 at cell tips.

Another significant difference between fission yeast and budding yeast is that in fission yeast, interphase microtubules (MTs) make important contributions to cell polarity regulation (Huffaker et al., 1988; Jacobs et al., 1988; Martin and Arkowitz, 2014; Chiou et al., 2017). In this regard, fission yeast is likely more similar to mammalian cells, in which MTs can interact directly or indirectly with multiple polarity regulators and also provide tracks for directed transport of vesicles and signaling molecules (Etienne-Manneville, 2013; Neukirchen and Bradke, 2011; Siegrist and Doe, 2007; Sugioka and Sawa, 2012). Interphase MTs in fission yeast are nucleated from multiple intracellular sites and form three to five bundles, each containing two to five MTs, that extend along the long axis of the cell (Chang and Martin, 2009; Sawin and Tran, 2006). Landmark proteins such as Tea1 and Tea4 are continuously delivered to the cell tip via the plus ends of dynamic MTs (Martin et al., 2005; Mata and Nurse, 1997; Tatebe et al., 2005). Landmark proteins further recruit polarity factors such as the protein kinase Pom1, PP1 protein phosphatase Dis2, formin For3 and actin-associated protein Bud6 (Alvarez-Tabares et al., 2007; Bahler and Pringle, 1998; Glynn et al., 2001; Martin et al., 2005).

The importance of MTs in fission yeast cell polarity has been demonstrated by pharmacological inhibition (Sawin and Nurse, 1998; Sawin and Snaith, 2004), and by mutation of genes involved in microtubule biogenesis and function (Hirata et al., 1998; Radcliffe et al., 1998; Umesono et al., 1983; Vardy and Toda, 2000; Anders et al., 2006; Samejima et al., 2005; Sawin et al., 2004). Mutations affecting MT nucleation and organization often lead to curved cells, whereas mutations affecting landmark proteins tend to lead to bent or branched cells, particularly after stress. By contrast, mutations in the Cdc42 polarity module lead to round- or wide-cell phenotypes (Chang et al., 1994; Kelly and Nurse, 2011; Miller and Johnson, 1994). Collectively, these findings have led to the view that MTs and MT-dependent landmark proteins are important for selecting sites of polarity establishment, but not for polarity establishment per se or maintenance of polarized growth (Chang and Martin, 2009; Sawin and Snaith, 2004). The differences in phenotypes mentioned above (i.e. mispositioned polarity versus lost or impaired polarity) further highlight our limited understanding of how MTs and MT-dependent landmarks contribute to regulation of Cdc42-dependent cell polarity.

Here, we address the question of how the two fission yeast Cdc42 GEFs are coordinated in cell polarity regulation, and how MTs and their effectors contribute to regulation of the core cell polarity machinery. Previous work showed that although *scd1*Δ cells are wide and/or round, they are nevertheless polarized during interphase (Kelly and Nurse, 2011). Here, we find that polarized growth of *scd1*Δ cells, unlike wild-type cells, absolutely requires interphase MTs: after MT disruption, *scd1*Δ cells grow in an isotropic-like manner. We show that MTs promote polarized growth in *scd1* mutants via a pathway involving polarity proteins Tea1, Tea4 and Pom1 (the Tea1–Tea4–Pom1 ‘axis’), as well as Cdc42 GAP Rga4 (Das et al., 2007; Kokkoris et al., 2014; Tatebe et al., 2008). Remarkably, this pathway serves to counteract the activity of Gef1, which, contrary to some previous reports (Das et al., 2009, 2015; Kokkoris et al., 2014; Vjestica et al., 2013), we find to be a cytosolic ‘global’ Cdc42 GEF rather than a membrane-associated ‘local’ GEF like Scd1. Our results reveal a previously unrecognized role for MTs and the Tea1–Tea4–Pom1 axis in the maintenance of fission yeast cell polarity, and they suggest a model in which local and global Cdc42 GEFs are active in parallel but regulated by different mechanisms. If not coordinated, these can impair rather than promote polarized growth.

## RESULTS

### Polarized growth of *scd1Δ* cells

Previously it was shown that hydroxyurea (G1/S phase)-arrested *scd1*Δ cells have a polarized shape (Kelly and Nurse, 2011). This suggested that *scd1*Δ cells are normally polarized, but, because of their round shape, this polarization can be observed unambiguously only during extended interphase. To investigate polarization of *scd1*Δ without using hydroxyurea, we overexpressed the CDK inhibitory knase Wee1 (*adh13:wee1*) in *scd1*Δ cells that also expressed CRIB-3mCitrine, a reporter for active (GTP-bound) Cdc42 (Jaquenoud and Peter, 2000; Mutavchiev et al., 2016; Tatebe et al., 2008). Compared with *scd1*Δ cells, *adh13:wee1 scd1*Δ cells were clearly polarized, although also wider than wild-type cells (Fig. S1). Interestingly, in spite of this polarization, we did not detect CRIB-3mCitrine at cell tips in *adh13:wee1 scd1*Δ cells (Fig. S1A), similar to observations of hydroxyurea-arrested *scd1*Δ cells (Kelly and Nurse, 2011).

**Fig. 1.**
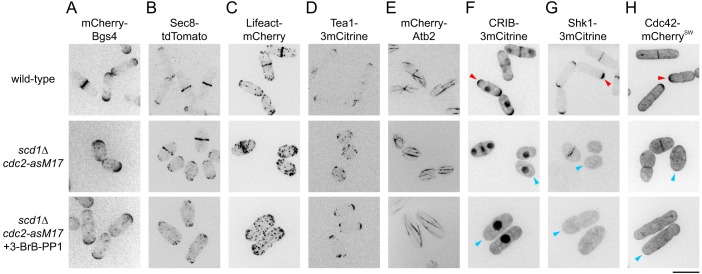
**Polarized growth of *scd1*Δ cells during extended interphase.** (A-H) Cell morphology and localization of polarity-associated proteins in cells of the indicated genotypes. 3-BrB-PP1 was added 5 h before imaging to inhibit analog-sensitive Cdc2 (bottom row). Arrowheads in F and G indicate detection (red) or no significant detection (blue) at cell tips. Arrowheads in H indicate enrichment (red) or no enrichment (blue) at tips. Scale bar: 10 µm. See also Fig. S1.

To characterize *scd1*Δ polarized growth in further detail, we used *cdc2-asM17* cells, which have a mutation in the ATP-binding pocket of Cdc2 and can be arrested in interphase by treatment with nucleotide-competitive analogs (Aoi et al., 2014; Bishop et al., 2000; Cipak et al., 2011). We imaged several different fluorescent-tagged cell polarity reporters in *scd1*Δ *cdc2-asM17* cells (Fig. 1). After treatment with the nucleotide-competitive analog 4-amino-1-tert-butyl-3-(3-bromobenzyl)pyrazolo[3,4-d]pyrimidine (3-BrB-PP1), *scd1*Δ *cdc2-asM17* cells were clearly polarized, and beta-glucan synthase Bgs4 (Cortés et al., 2005, 2015), exocyst component Sec8 (Snaith et al., 2011; Wang et al., 2002), F-actin reporter Lifeact (Huang et al., 2012; Riedl et al., 2008) and polarity landmark Tea1 (Mata and Nurse, 1997) were all localized to cell tips, as in wild-type cells (Fig. 1A-E). By contrast, CRIB, polarity kinase Shk1 (Qyang et al., 2002) and Cdc42 itself (Bendezú et al., 2015) were either not detected (CRIB, Shk1) or not visibly enriched (Cdc42) at cell tips after the same treatment (Fig. 1F-H).

We conclude that *scd1*Δ cells can grow in a polarized manner, with nearly all of the hallmarks of normal polarized growth. Owing to the increased width of *scd1*Δ cells, their polarized growth is most easily apparent during extended interphase. In addition, polarized growth in *scd1*Δ is not associated with detectable levels of the CRIB reporter or Shk1 at cell tips (Kelly and Nurse, 2011; see Discussion).

### Polarized growth in *scd1* mutants depends on microtubules and on polarity landmark proteins Tea1 and Tea4

**Fig. 2.**
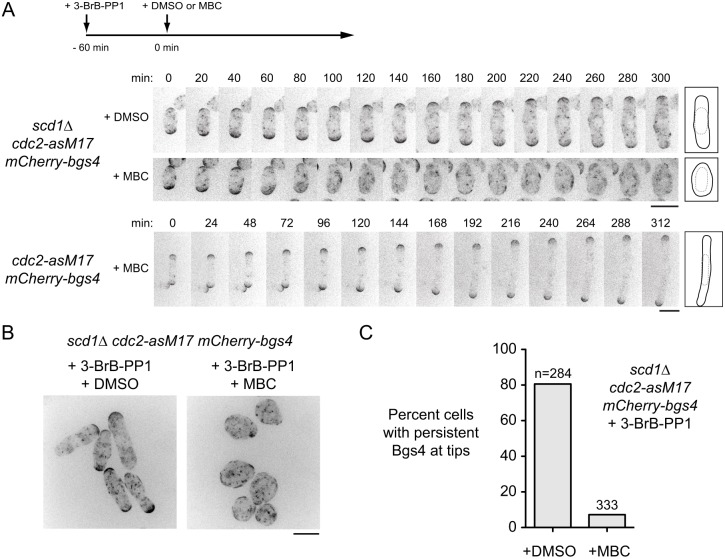
**Microtubule depolymerization in *scd1*Δ cells leads to PORTLI growth.** (A) Movie timepoints showing cell morphology and mCherry-Bgs4 distribution in the indicated genotypes, treated with 3-BrB-PP1 at −60 min and then with DMSO or MBC (plus 3-BrB-PP1). Diagrams show outlines at the beginning and end of movies. (B) Fields of cells as in A, after 3-BrB-PP1 and DMSO or MBC treatment for 300 min. (C) Quantification of mCherry-Bgs4 at cell tips during DMSO or MBC treatment (see Materials and Methods); ‘n’ indicates the number of cells scored. The difference between DMSO and MBC treatment was highly significant (*P*<0.0001; Fisher's exact test). Scale bars: 10 µm. See also Movie 1.

Inability to detect CRIB-3mCitrine at cell tips in *scd1*Δ cells led us to ask what other factors might be important for *scd1*Δ polarized growth. Although MTs are not required for polarized growth in wild-type (*scd1+*) cells (Sawin and Snaith, 2004), we hypothesized that MTs might contribute specifically to polarized growth in *scd1*Δ cells. We imaged mCherry-Bgs4 in *scd1*Δ *cdc2-asM17* cells during extended interphase after 3-BrB-PP1 treatment, both in the presence and absence of the MT-depolymerizing drug methyl-2-benzimidazole carbamate (MBC) (Fig. 2; Movie 1). Inhibition of Cdc2-asM17 allowed imaging of cell growth for several hours without intervening cell division. In the absence of MBC, *scd1*Δ *cdc2-asM17* grew in a polarized manner, as did control (*scd1+*) *cdc2-asM17* cells in the presence of MBC. Strikingly, after addition of MBC to *scd1*Δ *cdc2-asM17* cells, Bgs4 no longer localized mainly to cell tips and instead formed transient, mobile patches on the plasma membrane (Fig. 2A). Accordingly, instead of growing in a polarized manner, MBC-treated cells became increasingly round over time (Fig. 2B,C). Although average growth in these cells appeared to be isotropic, because of the dynamic, nonuniform distribution of Bgs4 on the plasma membrane we will refer to this growth pattern as ‘polarity transience leading to isotropic-like’ (PORTLI) growth. We conclude that MTs are crucial for polarized growth in *scd1*Δ cells, but not in wild-type (*scd1+*) cells*.*

**Fig. 3.**
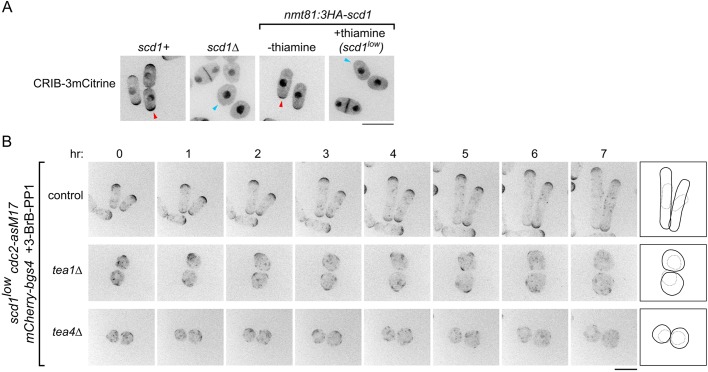
**When *scd1* is expressed at very low levels, *tea1*Δ and *tea4*Δ cells show PORTLI growth.** (A) Cell morphology and CRIB-3mCitrine localization in the indicated genotypes. Thiamine represses *nmt81:3HA-scd1* expression (‘*scd1^low^*’). Arrowheads indicate detection (red) or no significant detection (blue) of CRIB-3mCitrine at tips. (B) Movie timepoints showing cell morphology and mCherry-Bgs4 distribution in the indicated genotypes. *scd1* expression was repressed 24 h before imaging. 3-BrB-PP1 was added 30 min before imaging. Diagrams show outlines at the beginning and end of movies. Scale bars: 10 µm. See also Movie 2.

We hypothesized that MTs might contribute to polarized growth in *scd1*Δ cells via the landmark proteins Tea1 and Tea4. Interestingly, and consistent with this hypothesis, *tea1*Δ *scd1*Δ double mutants are inviable (Papadaki et al., 2002). Therefore, to construct double mutants of *scd1* with *tea1*Δ and *tea4*Δ, we generated a strain in which expression of 3HA-tagged Scd1 is controlled by the weak, thiamine-repressible *nmt81* promoter (Basi et al., 1993) (Fig. 3A). For simplicity, we will refer to the repressed *nmt81:3HA-scd1* allele as *scd1^low^*. Under repressing conditions, *scd1^low^* cells had a round morphology and lacked detectable CRIB-3mCitrine at cell tips. We note, however, that other mutant phenotypes (see below) indicate that some biologically relevant, functional Scd1 is produced in these cells, albeit at very low levels.

**Fig. 4.**
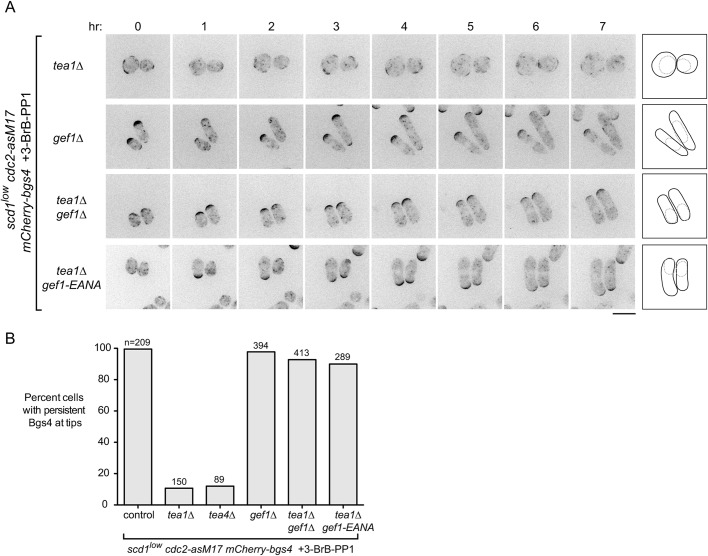
**Loss of *gef1* function restores polarized growth to *scd1^low^ tea1*Δ cells.** (A) Movie timepoints showing cell morphology and mCherry-Bgs4 distribution in the indicated genotypes. *scd1* expression was repressed 24 h before imaging. 3-BrB-PP1 was added 30 min before imaging. Diagrams show outlines at the beginning and end of movies. Note that newborn daughter cells often have less mCherry-Bgs4 at cell tips. (B) Quantification of mCherry-Bgs4 at cell tips, from movies of the type in Figs 3 and 4A; ‘n’ indicates the number of cells scored. Pairwise differences relative to control (first column) were highly significant for all strains except *gef1*Δ, (*P*<0.0001; Fisher's exact test, with correction for multiple comparisons). Scale bar: 10 µm. See also Fig. S3 and Movie 4.

We introduced *tea1*Δ and *tea4*Δ mutations into *scd1^low^ mCherry-Bgs4 cdc2-asM17* backgrounds. Under repressing conditions, *tea1*Δ *scd1^low^ mCherry-Bgs4 cdc2-asM17* and *tea4*Δ *scd1^low^ mCherry-Bgs4 cdc2-asM17* were viable but showed slightly increased frequency of cell death (see Materials and Methods). We repressed Scd1 expression for 24 h and then imaged cells after 3-BrB-PP1 addition (Fig. 3B; Movie 2). In control 3-BrB-PP1-treated cells, mCherry-Bgs4 remained highly polarized at cell tips, and cells grew in a polarized manner. By contrast, in *tea1*Δ and *tea4*Δ backgrounds even before 3-BrB-PP1 addition, cells were round, and mCherry-Bgs4 was present on the plasma membrane as small, randomly positioned patches (sometimes barely detectable) and on internal membranes. After 3-BrB-PP1 addition, *tea1*Δ *scd1^low^ mCherry-Bgs4 cdc2-asM17* and *tea4*Δ *scd1^low^ mCherry-Bgs4 cdc2-asM17* showed PORTLI growth, with transient, mobile mCherry-Bgs4 patches (Figs 3B and 4B; Movie 2). This indicates that when Scd1 is expressed at very low levels, the absence of either Tea1 or Tea4 leads to loss of normal polarity. We further confirmed these results by imaging exponentially growing *scd1^low^* and *scd1^low^ tea1*Δ cells in *cdc2+* backgrounds (Fig. S2, Movie 3).

### *gef1* loss of function relieves the requirement for Tea1 and Tea4 in *scd1^low^* polarized growth

We next tested whether Gef1 contributes to polarized growth when *scd1* function is compromised (Fig. 4). Because *gef1*Δ *scd1*Δ double mutants are inviable, we introduced *gef1*Δ into *scd1^low^ mCherry-Bgs4 cdc2-asM17* cells. Under repressing conditions, *gef1*Δ *scd1^low^ mCherry-Bgs4 cdc2-asM17* cells remained viable. Moreover, mCherry-Bgs4 was strongly enriched at cell tips both before and after 3-BrB-PP1 addition, and cells grew in a highly polarized manner (Fig. 4A,B; Movie 4). These results indicate that Gef1 is not required for polarized growth in *scd1^low^* cells and, thus, that the very low level of Scd1 expressed in *scd1^low^* cells is sufficient for viability and polarized growth. This in turn raised the question of why Tea1 and Tea4 are required for polarized growth in *scd1^low^* cells.

We hypothesized two possible roles for the Tea1/Tea4 system. The first possibility was that Tea1 and Tea4 might enhance the intrinsic ability of Scd1 to serve as a GEF when expressed at very low levels. The second possibility, which was motivated by the observation that Gef1 overexpression causes cell rounding (Coll et al., 2003; Das et al., 2012), was that rather than supporting Scd1 function directly, Tea1 and Tea4 might prevent or counteract any inappropriate function of Gef1, which would otherwise somehow interfere with the ability of low levels of Scd1 to promote polarized growth.

To distinguish between these possibilities, we introduced *gef1*Δ into *tea1*Δ *scd1^low^ mCherry-Bgs4 cdc2-asM17* cells and imaged cells after *scd1* repression and 3-BrB-PP1 addition. Remarkably, *gef1*Δ completely reversed the PORTLI growth of *tea1*Δ *scd1^low^ mCherry-Bgs4 cdc2-asM1*7 cells, which now grew in a highly polarized manner (Fig. 4A,B; Movie 4). We obtained qualitatively similar results without Cdc2 inhibition (i.e. in the absence of 3-BrB-PP1; Fig. S3A). These results provide strong support for the second of the two possible roles proposed above.

In addition to a central catalytic Dbl homology (DH) domain required for GEF activity, Gef1 contains an N-terminal region of unknown function and a C-terminal region that is proposed to contain a Bin/amphiphysin/Rvs (BAR) domain (Das et al., 2015). Because *gef1*Δ abolishes expression of the entire Gef1 protein, it remained unclear whether the polarized growth seen in *gef1*Δ *tea1*Δ *scd1^low^ mCherry-Bgs4 cdc2-asM1*7 cells was specifically caused by loss of Gef1 GEF activity. We therefore mutated conserved residues E318 and N505 in the Gef1 DH domain to generate a mutant (*E318A*, *N505A*; termed *gef1-EANA*) that, based on previous structural and *in vitro* biochemical analyses, should fold properly but fail to bind Cdc42 (Aghazadeh et al., 1998; Rossman et al., 2002a,b, 2005). Consistent with this, we found that *gef1-EANA* is a loss-of-function allele, even though Gef1-EANA protein localized *in vivo* identically to wild-type Gef1 (Fig. S3B-E) (Wei et al., 2016). In further imaging experiments, we found that after *scd1* repression, *gef1-EANA tea1*Δ *scd1^low^ mCherry-Bgs4 cdc2-asM1*7 cells were polarized both before and after 3-BrB-PP1 addition (Fig. 4A,B; Movie 4). This indicates that the reversal of PORTLI growth seen in our experiments can be attributed specifically to the loss of Gef1 GEF activity, rather than to the absence of Gef1 protein more generally.

Collectively, these results suggest not only that Gef1 is not required for polarized growth in *scd1^low^* cells but also that preventing or counteracting Gef1 activity is a prerequisite for polarized growth in *scd1^low^* cells. According to this view, the main role of Tea1 (and Tea4) in promoting polarized growth in *scd1^low^* cells is to prevent PORTLI growth caused by inappropriate Gef1 activity, because if Gef1 is not present, then Tea1 is no longer required for polarized growth.

### During unperturbed interphase, Gef1 is cytosolic rather than membrane associated

How is Gef1 localized *in vivo* such that it can promote PORTLI growth in *scd1^low^* cells? Initial characterization of Gef1 showed that it localized to the septum during cell division but did not have any specific localization during interphase (Coll et al., 2003; Hirota et al., 2003). However, it was later reported that Gef1 is also localized to cell tips during interphase (Das et al., 2009, 2015; Kokkoris et al., 2014; Vjestica et al., 2013). Because it was not obvious to us how cell tip-localized Gef1 would lead to PORTLI growth, we reinvestigated Gef1 interphase localization.

**Fig. 5.**
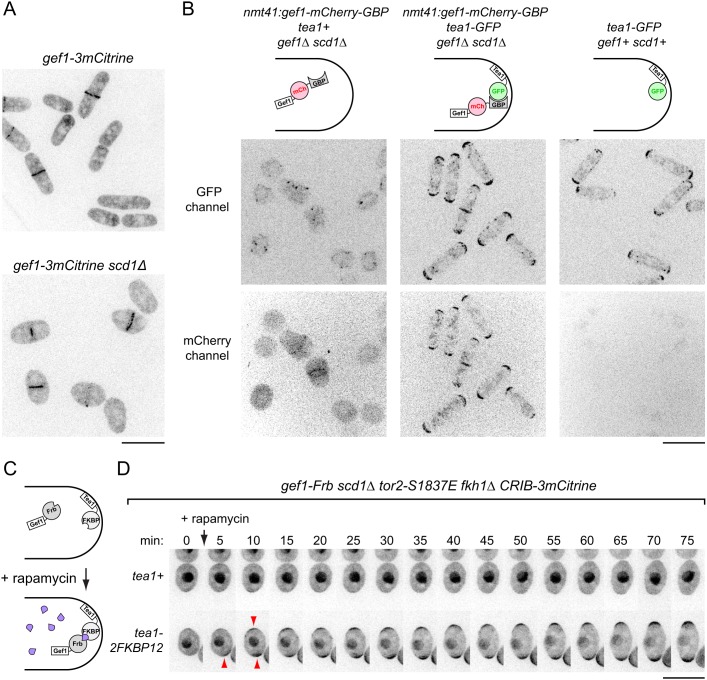
**Gef1 is normally cytosolic and is active during interphase, as targeting Gef1 to cell tips in *scd1*Δ cells restores wild-type morphology and Cdc42-GTP enrichment at tips.** (A) Localization of Gef1-3mCitrine in wild-type and *scd1*Δ cells. In both cases, Gef1 is present at the septum but not at cell tips. (B) Ectopic targeting of Gef1-mCherry-GBP to cell tips by coexpression of Tea1-GFP, in *scd1*Δ background. In untagged *tea1+* cells (left), Gef1-mCherry-GBP remains cytosolic, and cells are round. In *tea1-GFP* cells (middle), Gef1-mCherry-GBP is at cell tips, and cells are polarized. Right panels show absence of bleed through from the GFP channel to the mCherry channel. (C) Schematic of targeting Gef1 to cell tips by rapamycin-induced dimerization. (D) CRIB-3mCitrine localization after time-resolved targeting of Gef1 to cell tips by rapamycin-induced dimerization with Tea1-2FKBP12. Negative control cells express untagged Tea1 (*tea1+*). Rapamycin was added just after the 0 min timepoint. Arrowheads indicate appearance of CRIB-3mCitrine at tips after rapamycin addition. Scale bars: 10 µm. See also Figs S4 and S5 and Movies 5 and 6.

Using several different fluorescent Gef1 fusion proteins, including previously published ones, we observed Gef1 at the septum during cell division, but did not observe any specific localization of Gef1 during interphase, even with sensitive detection (Fig. 5A; Fig. S4A). We also did not observe specific localization of Gef1 in interphase *scd1*Δ cells (Fig. 5A,B), and we confirmed that our method of preparing cells for imaging does not introduce artifacts (Fig. S4B; see Materials and Methods). These results indicate that Gef1 is normally cytosolic and not enriched on the plasma membrane during interphase.

Interestingly, however, we did find conditions under which Gef1 becomes localized to cell tips. We treated cells expressing Gef1-3YFP with thiabendazole (TBZ), a drug that not only depolymerizes MTs but also leads to a stress that depolarizes the actin cytoskeleton for 60–90 min, via a non-MT-related mechanism (Sawin and Nurse, 1998; Sawin and Snaith, 2004). Upon TBZ treatment, Gef1-3YFP transiently localized to cell tips before becoming associated with mobile patches on the plasma membrane on cell sides (Fig. S4C,D; Movie 5). As we have recently found that stress signaling regulates the Cdc42 cell polarity module (Mutavchiev et al., 2016), we speculate that during some imaging protocols, it is possible that some form of mild unintended stress could cause cytosolic Gef1 to associate with the plasma membrane at cell tips (see Discussion).

### Targeting to cell tips converts Gef1 from a global to a local Cdc42 GEF

Together with our finding that *gef1*Δ and *gef1-EANA* mutations restore polarized growth to *tea1*Δ *scd1^low^* cells, our observation that Gef1 is normally cytosolic suggested that the PORTLI growth seen in *scd1* mutants in the presence of MBC or in *tea1*Δ or *tea4*Δ backgrounds is caused by Gef1 acting on membrane-associated Cdc42 from a cytosolic pool, as a ‘global’ Cdc42 GEF. To support this view, we asked whether artificial targeting of Gef1 to cell tips – that is, changing a ‘global’ Cdc42 GEF into a ‘local’ GEF – would convert it from a promoter of PORTLI growth into a promoter of polarized growth.

In one set of experiments, we used GFP and GFP-binding protein (GBP) (Rothbauer et al., 2008) to heterodimerize Gef1 with Tea1 (Fig. 5B). Fusion of Gef1-mCherry to GBP rescued the synthetic lethality of *gef1*Δ *scd1*Δ cells, indicating that Gef1-mCherry-GBP is functional. In *gef1*Δ *scd1*Δ cells expressing untagged Tea1, Gef1-mCherry-GBP was cytosolic during interphase, and cells displayed the wide or round morphology expected for *scd1*Δ mutants*.* By contrast, in *gef1*Δ *scd1*Δ cells expressing Tea1-GFP, which is normally localized to cell tips (Behrens and Nurse, 2002), Gef1-mCherry-GBP relocalized from the cytosol to cell tips, and cells displayed a normal, wild-type morphology. This demonstrates that targeting Gef1 to cell tips is sufficient to promote highly robust polarized growth in *scd1*Δ cells.

In a second set of experiments, we used rapamycin-induced dimerization (Chen et al., 1995; Haruki et al., 2008) to target Gef1 to cell tips (Fig. 5C). We tagged Gef1 with an FKBP-rapamycin-binding (Frb) domain, and Tea1 with 2×12kD-FK506- and rapamycin-binding protein (2FKBP12) (Ding et al., 2014). Because this does not require GFP-tagging of Gef1 or its dimerization partner, it allowed us to image CRIB-3mCitrine as a reporter of the Cdc42 cell polarity module. We first validated dimerization by replacing endogenous Gef1 and Tea1 with Gef1-Frb-GFP and Tea1-2FKBP12 fusion proteins, in a *scd1*Δ background. Upon rapamycin addition, Gef1-Frb-GFP was rapidly recruited from the cytosol to cell tips, and cells became more polarized (Fig. S5A; Movie 6). We then replaced Gef1 and Tea1 with Gef1-Frb (i.e. without GFP) and Tea1-2FKBP12 in a *scd1*Δ *CRIB-3mCitrine* background. Before rapamycin addition, interphase cells showed nearly undetectable levels of CRIB-3mCitrine at cell tips. However, upon addition of rapamycin, CRIB-3mCitrine quickly appeared at cell tips, and morphology and polarized growth became similar to that of wild-type cells (Fig. 5D; Fig. S5B,C). By contrast, in control cells expressing Gef1-Frb, rapamycin did not induce CRIB-3mCitrine localization to cell tips. Taken together, these results indicate that relocalizing Gef1 from the cytosol to cell tips converts it from a global to a local Cdc42 GEF.

### Pom1 kinase activity is required for polarized growth of *scd1Δ* cells

**Fig. 6.**
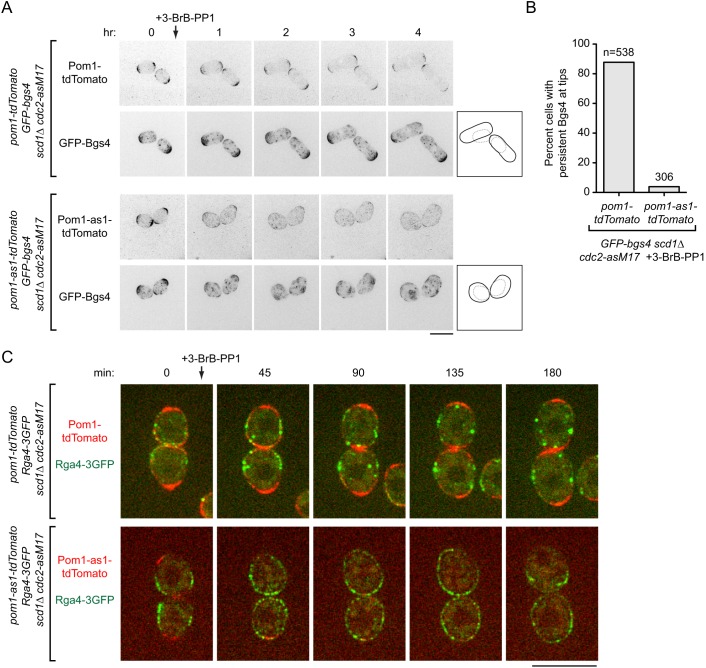
**Inhibition of Pom1 kinase activity in *scd1*Δ cells leads to PORTLI growth and randomized localization of Cdc42 GAP Rga4.** (A) Movie timepoints showing cell morphology and distribution of Pom1-tdTomato or Pom1-as1-tdTomato and GFP-Bgs4 in the indicated genotypes after 3-BrB-PP1 treatment (added just after the 0 h timepoint). 3-BrB-PP1 inhibits both Cdc2-asM17 and Pom1-as1-tdTomato. Diagrams show outlines at the beginning and end of movies. (B) Quantification of GFP-Bgs4 at cell tips, from movies of the type in A. Differences were highly significant (*P*<0.0001; Fisher's exact test). (C) Single focal-plane movie timepoints showing cell morphology and distribution of Pom1-tdTomato or Pom1-as1-tdTomato and Rga4-3GFP in the indicated genotypes after 3-BrB-PP1 treatment. Scale bars: 10 µm. See also Figs S6 and S7 and Movies 7 and 8.

To understand how MTs, Tea1 and Tea4 might counteract Gef1 to allow polarized growth in *scd1* mutants, we investigated the polarity protein kinase Pom1 (Bahler and Pringle, 1998). Pom1 is localized to the plasma membrane and enriched at cell tips, and this depends both on Tea1 and Tea4 and on Pom1 kinase activity (Hachet et al., 2011). We introduced the analog-sensitive allele *pom1-as1-tdTomato* (Hachet et al., 2011), or control *pom1-tdTomato*, into *scd1*Δ *GFP-Bgs4 cdc2-asM17* cells, and used 3-BrB-PP1 to simultaneously inhibit analog-sensitive Pom1 and Cdc2 (Fig. 6A,B; Fig. S6A, Movie 7). In control *pom1-tdTomato* cells, GFP-Bgs4 and Pom1-tdTomato localized to cell tips both before and after 3-BrB-PP1 addition, and cells grew in a polarized manner. In *pom1-as1-tdTomato* cells, GFP-Bgs4 and Pom1-as1-tdTomato localized to cell tips before 3-BrB-PP1 addition, but after 3-BrB-PP1 addition, both proteins became delocalized, and cells showed PORTLI growth. This demonstrates that Pom1 kinase activity is required for polarized growth of *scd1*Δ cells.

To determine whether PORTLI growth after Pom1 inhibition depends on Gef1, we introduced either a *pom1*Δ single mutation or *pom1*Δ *gef1*Δ double mutation into *scd1^low^ mCherry-Bgs4 cdc2-asM17* cells. After 3-BrB-PP1 addition, *pom1*Δ *scd1^low^ mCherry-Bgs4 cdc2-asM17* showed PORTLI growth, while *pom1*Δ *gef1*Δ *scd1^low^ mCherry-Bgs4 cdc2-asM17* grew in a polarized manner (Fig. S6B, Movie 8). These results suggest that Pom1, like Tea1 and Tea4, contributes to polarized growth of *scd1* mutant cells by counteracting Gef1.

One role of Pom1 is to regulate localization of the Cdc42 GTPase activating protein (GAP) Rga4 (Das et al., 2007; Tatebe et al., 2008). In wild-type cells, Rga4 is localized to the plasma membrane and enriched on cell sides but excluded from cell tips. By contrast, in *pom1*Δ and *pom1* kinase-inactive mutants, Rga4 is no longer excluded from nongrowing cell tips (Tatebe et al., 2008). We therefore examined Rga4-3GFP localization in *pom1-as1-tdTomato* and *pom1-tdTomato* cells in *scd1*Δ *cdc2-asM17* backgrounds after 3-BrB-PP1 addition (Fig. 6C; Fig. S7). In control *pom1-tdTomato scd1*Δ *cdc2-asM17* cells, Rga4-3GFP remained largely excluded from cell tips. By contrast, in *pom1-as1-tdTomato scd1*Δ *GFP-Bgs4 cdc2-asM17* cells, Rga4-3GFP quickly became much more uniformly distributed on the plasma membrane, coincident with redistribution of Pom1-as1-tdTomato and the onset of PORTLI growth.

### Cdc42 GAP Rga4 counteracts Gef1-dependent PORTLI growth

In principle, the more uniform distribution of Rga4-3GFP after Pom1 inhibition could be either a consequence or a cause of PORTLI growth. To distinguish between these possibilities, we investigated how Rga4 contributes to polarized growth when *scd1* function is compromised, and how this is affected by Gef1.

**Fig. 7.**
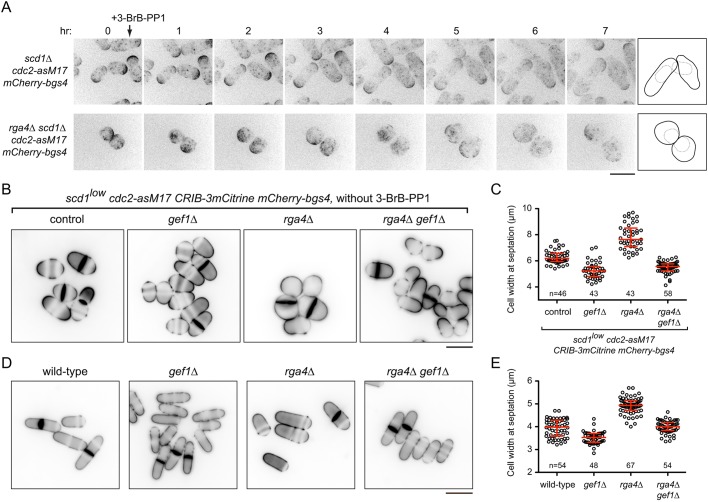
***scd1*Δ *rga4*Δ cells show PORTLI growth, and *gef1*Δ rescues the short/wide-cell phenotypes associated with *rga4*Δ.** (A) Movie timepoints showing cell morphology and mCherry-Bgs4 distribution in the indicated genotypes after 3-BrB-PP1 treatment (added just after the 0 h timepoint). Diagrams show outlines at the beginning and end of movies. Outlines are more obvious in movies (Movie 9). (B) Calcofluor staining of actively cycling cells for the indicated genotypes. *scd1* expression was repressed 24 h before imaging. Although *cdc2-asM17* is present, 3-BrB-PP1 was not added to cultures. (C) Cell width at septation for the genotypes in B. Median and interquartile ranges are shown. All pairwise comparisons were highly significant (Mann–Whitney test; *P*<0.0001 for all except *gef1*Δ versus *rga4*Δ *gef1*Δ, for which *P*=0.004). ‘n’ indicates the number of cells scored. (D) Calcofluor staining of actively cycling wild-type cells and mutants indicated, in wild-type background. (E) Cell width at septation for the genotypes in D. Median and interquartile ranges are shown. All pairwise comparisons were highly significant (Mann–Whitney test; *P*<0.0001), except wild-type versus *rga4*Δ *gef1*Δ (*P*=0.57). Scale bars: 10 μm. See also Fig. S8 and Movie 9.

We first analyzed *rga4*Δ *scd1*Δ double mutants. Previous single-time-point images indicated that *rga4*Δ *scd1*Δ double mutants are especially wide (Kelly and Nurse, 2011) and, after hydroxyurea arrest, nearly round (Revilla-Guarinos et al., 2016). We introduced *rga4*Δ into *scd1*Δ *cdc2-asM17 mCherry-Bgs4* cells and imaged cell growth over several hours after 3BrB-PP1 addition. In contrast to the polarized growth of *scd1*Δ *cdc2-asM17* cells, *rga4*Δ *scd1*Δ *cdc2-asM17* cells showed PORTLI growth, with transient, mobile patches of mCherry-Bgs4 on the plasma membrane (Fig. 7A; Movie 9).

To investigate the role of Gef1 in Rga4-dependent polarized growth, we generated *rga4*Δ and *rga4*Δ *gef1*Δ mutants in a *scd1^low^ cdc2-asM17* background and analyzed them both with and without 3-BrB-PP1 (these cells also expressed CRIB-3mCitrine and Bgs4-mCherry) (Fig. S8). During extended interphase after 3-BrB-PP1 addition, *rga4*Δ *scd1^low^ cdc2-asM17* cells were compromised in polarity, becoming wider and rounder than control *scd1^low^ cdc2-asM17* cells. Although these polarity defects were not as extreme as in *rga4*Δ *scd1*Δ *cdc2-asM17* or *tea1*Δ *scd1^low^ cdc2-asM17* cells under similar conditions, they were almost completely rescued by additional deletion of *gef1* (Fig. S8; see Discussion). During exponential growth (i.e. without 3-BrB-PP1), *rga4*Δ *scd1^low^ cdc2-asM17* cells were also significantly wider than isogenic control (*rga4+*) cells, and this was also rescued by the additional deletion of *gef1* (Fig. 7B,C). Collectively, these results indicate that polarity defects associated with *rga4*Δ in *scd1* mutants are mediated through Gef1.

Rescue of *rga4*Δ polarity defects by *gef1*Δ in a *scd1^low^* background appeared to conflict with a previous report that *rga4*Δ *gef1*Δ double mutants were wider than either *rga4*Δ or *gef1*Δ single mutants (Kelly and Nurse, 2011). We therefore reinvestigated cell dimensions of *rga4*Δ and *gef1*Δ single and double mutants in a fully wild-type background (Fig. 7D,E). Consistent with an earlier characterization (Das et al., 2007), *rga4*Δ cells were wider than wild-type cells. However, we also found that additional deletion of *gef1* restored *rga4*Δ cells to normal width. Our results in a wild-type (*scd1+*) background thus contradict previous work (Kelly and Nurse, 2011) and suggest that increased width of *rga4*Δ (*scd1+*) cells is a consequence of global Gef1 activity competing, albeit with limited success, against relatively strong local Scd1 activity.

## DISCUSSION

### Cell polarity regulation by local and global Cdc42 GEFs

**Fig. 8.**
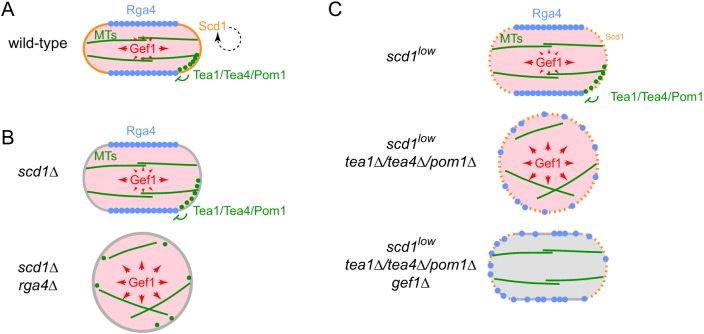
**Simplified schematic model of polarized growth via microtubule-dependent coordination of local and global Cdc42 GEF activities.** (A) In wild-type cells, five main features of the model lead to normal polarized growth: (1) Scd1 (orange) is a plasma membrane-associated ‘local’ Cdc42 GEF at cell tips and maintains a focused polarity zone via positive feedback; (2) Gef1 (pink) is a cytosolic, ‘global’ Gdc42 GEF; (3) microtubules (MTs; green) target the Tea1–Tea4–Pom1 axis (green) to cell tips; (4) this restricts Cdc42 GAP Rga4 (blue) to the plasma membrane at cell sides; (5) Rga4 on the membrane locally counters cytosolic Gef1 activity, preventing net GEF activity at cell sides (different-sized red arrows). (B) The model as applied to *scd1*Δ and *scd1*Δ *rga4*Δ cells. In *scd1*Δ cells, there is no strong focused polarity zone, but Rga4 can still locally counter global Gef1 activity, leading to greater ‘net’ Gef1 activity in the region of the cell tips, as in wild-type cells. Cells are therefore polarized but wider than wild-type. In *scd1*Δ *rga4*Δ cells, absence of Rga4 means that Gef1 is not locally countered anywhere and thus can promote PORTLI growth. Distribution of MTs and Tea1/Tea4/Pom1 will also be abnormal, owing to round cell shape. (C) The model as applied to the genotypes indicated. In *scd1^low^* cells, only a very limited amount of local Cdc42 GEF Scd1 is present at cell tips, and thus the polarity zone is not focused as in wild-type. However, ‘net’ Gef1 activity remains greater in the region of cell tips, and Gef1 cooperates with Scd1. In *scd1^low^ tea1*Δ/*tea4*Δ/*pom1*Δ cells, Rga4 is no longer spatially restricted, and therefore ‘net’ Gef1 activity is not spatially controlled. This competes with (low) Scd1 and overwhelms its contribution to polarized growth. In *scd1^low^ tea1*Δ/*tea4*Δ/*pom1*Δ *gef1*Δ cells, competition from Gef1 is alleviated, allowing the low Scd1 to support polarized growth.

Our results suggest a conceptual model for Cdc42- and MT-mediated cell polarity regulation in fission yeast (Fig. 8) that is significantly different from previous models (Chang and Martin, 2009; Hachet et al., 2012; Rincón et al., 2014; Sawin and Snaith, 2004; Chiou et al., 2017; Kokkoris et al., 2014; Martin and Arkowitz, 2014). Details of the model are presented in Fig. 8; we mention a few key points here.

We have shown that Gef1 is a cytosolic, ‘global’ Cdc42 GEF, unlike Scd1, which is a cell tip-localized, ‘local’ Cdc42 GEF (Hirota et al., 2003; Kelly and Nurse, 2011). Moreover, the functional outputs of these two GEFs are controlled by distinct mechanisms, working in parallel. Promotion of polarized growth by Scd1 is thought to be a direct consequence of its localization at cell tips, dependent on a positive feedback mechanism similar to that in budding yeast (Chiou et al., 2017; Endo et al., 2003; Kelly and Nurse, 2011; Woods and Lew, 2017). By contrast, the spatially uniform cytosolic distribution of Gef1 during interphase would allow it, in principle, to activate Cdc42 anywhere on the plasma membrane. However, global Gef1 activity is normally spatially antagonized by Cdc42 GAP Rga4, the localization of which is restricted to the cell sides by MTs and the Tea1–Tea4–Pom1 axis (Tatebe et al., 2008), leading to a ‘channeling’ of net Gef1 activity towards cell tips. The importance of restricting net Gef1 activity to cell tips is underscored by our finding that artificial targeting of Gef1 to cell tips in *scd1*Δ cells restores wild-type morphology and CRIB localization at tips.

We have shown that when *scd1* function is compromised, MTs and the Tea1–Tea4–Pom1 axis become essential for polarity maintenance. Previous work by us and others strongly supported the view that MTs and the Tea1–Tea4–Pom1 axis are important for specifying sites of cell polarity establishment, but not for polarity establishment per se, or polarity maintenance (Bahler and Pringle, 1998; Mata and Nurse, 1997; Chang and Martin, 2009; Martin et al., 2005; Sawin and Snaith, 2004; Tatebe et al., 2005). Our new results indicate that such a view is incomplete, and that a key role of the Tea1–Tea4–Pom1 axis is to counteract, via Rga4, any spatially inappropriate Gef1 activity at cell sides. In mammalian cells, there are similar examples of MTs regulating RhoGEF or RhoGAP distribution or activity, either directly or indirectly, in cell migration, cytokinesis and tissue organization (Birkenfeld et al., 2008; Etienne-Manneville, 2013; Meiri et al., 2012; Ratheesh et al., 2012; Siegrist and Doe, 2007; Yüce et al., 2005). Although not addressed in the current work, we note that MTs and the Tea1–Tea4–Pom1 axis are also important for new-end take-off (NETO), the transition from monopolar to bipolar growth (Bahler and Pringle, 1998; Martin et al., 2005; Mata and Nurse, 1997; Mitchison and Nurse, 1985; Nunez et al., 2016).

Our work further suggests that MTs provide the means for coordinating Gef1 function with Scd1 function. Normally, alignment of MTs along the long axis of the cell leads to positioning of MT-dependent landmarks at cell tips (Minc et al., 2009; Terenna et al., 2008) and therefore, ultimately, to enrichment of Rga4 at cell sides. Thus, when MTs and landmarks are present, the Scd1 and Gef1 systems cooperate to promote polarized growth at the same sites, i.e. the cell tips. By contrast, when MTs and/or landmarks are absent, the Scd1 (local) and Gef1 (global) systems can end up competing with each other, with Gef1 promoting PORTLI rather than polarized growth (e.g. in *scd1^low^ tea1*Δ).

Our model also provides new mechanistic interpretations of previously reported results. For example, *scd1*Δ and *rga4*Δ mutations were previously described as having additive effects on cell width, because the *scd1*Δ *rga4*Δ double mutant was found to be wider than either single mutant (Kelly and Nurse, 2011). However, our work demonstrates that the difference between the single mutants and the double mutant is in fact qualitative rather than quantitative, because while each single mutant is polarized, the *scd1*Δ *rga4*Δ double mutant shows PORTLI growth. Moreover, within the context of our model, the difference in cell shape between *scd1*Δ single mutants and *scd1*Δ *rga4*Δ double mutants, together with the rescue of *rga4*Δ phenotypes by *gef1*Δ, strongly suggests that the major physiological role of Rga4 in cell polarity regulation is to counteract the effects of Gef1.

### Polarized and PORTLI growth in cells with impaired Scd1 function

To analyze polarized growth in *scd1*Δ *and scd1^low^* cells, we imaged fluorescent-tagged beta-glucan synthase Bgs4, the localization of which normally correlates precisely with polarized growth (Cortés et al., 2005), and we extended interphase by inhibiting analog-sensitive Cdc2 (Aoi et al., 2014). Interestingly, during PORTLI growth, Bgs4 appears as transient and mobile patches on the plasma membrane instead of being distributed homogeneously. The transient nature of these patches will be interesting to investigate in the future.

Imaging during extended interphase allowed us to unambiguously identify growth patterns in *scd1* mutants, which normally do not elongate very much during a single cell cycle because of their short or wide shape. Extended interphase can also circumvent problems that arise if strains have abnormal phenotypes associated with cytokinesis (e.g. *pom1*Δ) (Bahler and Pringle, 1998; see Materials and Methods). Although there might be caveats to the use of analog-sensitive Cdc2, we observed similar differences in polarized versus PORTLI growth in several strains without Cdc2 inhibition; we therefore do not anticipate that Cdc2 inhibition significantly affects the overall interpretation of our results. In fission yeast, polarized growth continues when Cdc2 kinase is inactivated by either temperature- or analog-sensitive mutations (Dischinger et al., 2008; Nurse et al., 1976). In this context, fission yeast could be different from budding yeast, which has both polarized and isotropic growth periods during interphase, depending on the stage of bud formation (Chiou et al., 2017; Martin and Arkowitz, 2014). In the absence of inhibition, *cdc2-asM17* retains essentially all functionality of wild-type *cdc2+* (Aoi et al., 2014), unlike an earlier *cdc2-as* allele (Dischinger et al., 2008), and to inhibit Cdc2-asM17, we used the minimum concentration of analog required to prevent mitotic entry (see Materials and Methods). Under these conditions (i.e. in the absence of any other perturbations), both wild-type and *scd1*Δ cells show robust polarized growth.

While our initial experiments involved *scd1*Δ cells, many subsequent experiments involved *scd1^low^* cells. This was crucial for deciphering the relationship between Scd1, Gef1 and the Tea1–Tea4–Pom1 axis, because *scd1*Δ is synthetically lethal with *tea1*Δ and *gef1*Δ, whereas *scd1^low^* is not. At the same time, these differences in synthetic lethality highlight the fact that because *scd1^low^* cells retain some Scd1 function, they are not equivalent to *scd1*Δ cells. In particular, after 3-BrB-PP1 treatment (in *cdc2-asM17* backgrounds), *scd1*Δ *rga4*Δ cells show PORTLI growth, while *scd1^low^ rga4*Δ cells have less severe polarity defects (which are nevertheless rescued by *gef1*Δ). The simplest explanation for this is that in *scd1^low^ rga4*Δ cells, the polarity system set up by low levels of Scd1 can partially compete against the Gef1-dependent drive towards PORTLI growth. How low levels of Scd1 achieve this at a mechanistic level remains to be explored.

In this context, it is also interesting to compare polarity phenotypes of *scd1^low^ rga4*Δ with *scd1^low^ tea1*Δ, because *scd1^low^ tea1*Δ cells show more severe PORTLI growth (as do *scd1^low^ tea4*Δ, and *scd1^low^ pom1*Δ). We can imagine two nonexclusive explanations for this difference. First, in addition to regulating Rga4, the Tea1–Tea4–Pom1 axis could have a separate role in either bolstering *scd1^low^* function or countering *gef1* function. Tea1 was recently shown to have a role in limiting the distribution of sterol-rich membrane domains to cell poles (Makushok et al., 2016), via an unknown mechanism; however, it is unclear whether this could be important for polarized versus PORTLI growth, as *rga4*Δ *scd1*Δ cells are *tea1+* but still show PORTLI growth. Tea1 is also important for polarized growth of *for3*Δ cells (Feierbach et al., 2004) and in a *cdc42* allele with an added (engineered) transmembrane domain (Bendezú et al., 2015). Second, the different phenotypes could be caused by the presence versus the absence of Rga4. That is, in *scd1^low^ tea1*Δ cells, the GAP activity of Rga4 will be distributed essentially evenly over the entire plasma membrane, including at ‘prospective tip’ regions, thereby counteracting the weak polarizing activity of Scd1^low^; by contrast, in *scd1^low^ rga4*Δ cells, there is no Rga4 GAP activity anywhere, and therefore low levels of Scd1 could have a greater net effect on cell polarity.

Currently it is unclear why CRIB-3mCitrine is not detectable at cell tips in polarized *scd1*Δ and *scd1^low^* cells. Although it is formally possible that polarized growth in these cells does not involve GTP-bound Cdc42 at cell tips, it is equally plausible that the levels of GTP-bound Cdc42 and/or other factors required for CRIB reporter localization (Takahashi and Pryciak, 2007) are simply below the threshold necessary for detection. In this context, it is important to note that even though CRIB-3mCitrine is not detected at cell tips in *scd1^low^* cells, cell polarity phenotypes indicate that these cells nevertheless produce biologically important levels of Scd1.

### Gef1 localization during interphase

As we find that in unperturbed interphase cells, Gef1 is cytosolic, both in wild-type and in *scd1*Δ backgrounds, it is unclear why some (but not all) reports observed Gef1 at interphase cell tips (Das et al., 2015, 2009; Kokkoris et al., 2014; Vjestica et al., 2013). Our own results lead us to speculate that these reports could be due to unintended mild cell stress, possibly because of how cells are prepared for imaging, or because of phototoxicity during imaging (Laissue et al., 2017). In our experiments, cells are imaged under conditions that are essentially identical to those of cells growing in flasks, apart from shaking. This minimizes stress (Mutavchiev et al., 2016; see Materials and Methods) and allows imaging of polarized growth under the microscope for several hours.

Previous work has suggested that Gef1 is negatively regulated by phosphorylation via the NDR kinase Orb6 (Das et al., 2009, 2015); specifically, Orb6 is thought to prevent Gef1 from localizing to the plasma membrane on cell sides. Our results are not inconsistent with this view. However, because we find that Gef1 can be active as a cell-polarity GEF from the cytosol, we would argue that regulation of Gef1 membrane localization (specifically, to cell sides) is separable from regulation of Gef1 GEF activity per se. It is possible that localization of Gef1 to the plasma membrane on cell sides might further potentiate its net biological activity relative to any countering GAP activity from Rga4. These will be interesting questions to address in the future.

Regulated localization of Cdc42 GEFs to the plasma membrane could also be relevant to mammalian cells. Gef1 is unusual among RhoGEFs in that while it contains a catalytic DH domain, it lacks a pleckstrin homology (PH) domain, which is present in nearly all DH family RhoGEFs and is important for association with membrane lipids (Cook et al., 2014; Rossman et al., 2005). The mammalian Cdc42 GEF Tuba also lacks a PH domain and instead contains a BAR domain (Salazar et al., 2003); Gef1 has also been proposed to contain a BAR domain, although this has not been confirmed experimentally (Das et al., 2015). Interestingly, in MCDK epithelial cells, Tuba is localized to the cytoplasm when cells are grown in a monolayer, but is concentrated subapically when cells are grown to form cysts (Qin et al., 2010). Thus, like Gef1, the localization of Tuba might be subject to regulation, during development and/or differentiation.

### Links from polarity landmarks to Gef1 and Rga4

We showed previously that MT-based targeting of Tea1 to cell sides can promote new polarity axis formation, leading to branched cells (Sawin and Snaith, 2004). More recently, Kokkoris et al. reported that ectopically localized Tea4 can specify growth sites through a mechanism involving Gef1 and Rga4 (Kokkoris et al., 2014). These experiments were based on fusing an N-terminal Tea4 fragment (Tea4N) to the cortical node protein Cdr2 (Morrell et al., 2004; Wu et al., 2006), leading to localization of the Cdr2-Tea4N fusion protein to nodes at cell sides. The Cdr2-Tea4N fusion induced an ectopic ‘bulge’ at cell sides, and further experiments suggested that this was caused by local activation of Cdc42 via localized plasma membrane recruitment of Gef1 and exclusion of Rga4. Although both our current work and that of Kokkoris et al. (2014) suggest functional links from Tea4 to Gef1 and Rga4, there are several distinctions between the two studies. First, and most generally, the work of Kokkoris et al. suggests that the Tea4 landmark is ‘sufficient’ for growth at ectopic sites, whereas one aspect of our work has been to show that the Tea4 landmark (together with Tea1 and Pom1) is ‘necessary’ for polarized growth at normal cell tips, specifically when Scd1 function is compromised. Second, Kokkoris et al. reported that Gef1 is recruited to ectopic sites containing the Cdr2-Tea4N fusion. In contrast, we have shown that Gef1 is not detected on the plasma membrane of unperturbed wild-type or *scd1*Δ cells, although it can be enriched on the plasma membrane under certain conditions (e.g. TBZ treatment, independent of Tea4) (Fig. 5; Fig. S4, Movie 5). Third, the bulge induced by Cdr2-Tea4N did not require Pom1, whereas we find that Pom1 is essential for polarized growth in *scd1*Δ cells (Fig. 6; Figs S6 and S7, Movies 7 and 8). Fourth, bulging induced by Cdr2-Tea4N was dependent not only on Gef1 but also, surprisingly, on Rga4. In contrast, our data suggest that Rga4 on the plasma membrane at cell sides locally counteracts the effect of global Gef1 activity, thereby preventing growth in the cell middle. Finally, the ectopic bulge induced by Cdr2-Tea4N is qualitatively different from the conventional polarized growth seen at normal cell tips and in cells that establish a new polarity axis in the cell middle by other means (so-called ‘T’ shape) (Sawin and Snaith, 2004; Snell and Nurse, 1994). These differences suggest that the detailed mechanisms that lead to the Cdr2-Tea4N-induced ectopic bulge are distinct from those that polarize growth at a normal cell tip.

### Concluding remarks

What might be the purpose of regulating cell polarity by both local and global Cdc42 GEFs? Although here we can only speculate, we note that *gef1*Δ cells have a mild defect or delay in NETO (Coll et al., 2003; Das et al., 2012). Computational modeling suggests that the Gef1 contribution to total Cdc42 GEF activity could be an important feature in the timing of NETO and in the symmetry of Cdc42 activation at the two cell tips (Das et al., 2012). In light of our results, it could be of interest to investigate, in a more detailed spatial model, how the particular properties of a local versus a global GEF might influence the NETO transition. A second possible purpose relates to our observation that although Gef1 is cytosolic in unperturbed cells, it associates with the plasma membrane upon TBZ treatment (this work), as well as upon inhibition/inactivation of Orb6 (Das et al., 2009). Thus, Gef1 might have a specific role in regulating cell polarity in response to stress or cell signaling.

## MATERIALS AND METHODS

### Yeast culture

Standard fission yeast methods were used throughout (Forsburg and Rhind, 2006; Petersen and Russell, 2016). Growth medium was either YE5S rich medium (using Bacto yeast extract; Becton Dickinson) or PMG minimal medium, with glucose added after autoclaving [PMG is equivalent to Edinburgh minimal medium (EMM2) but uses 4 g/l sodium glutamate instead of ammonium chloride as a nitrogen source]. PMG was used only for experiments involving *scd1^low^* cells (i.e. *nmt81:3HA-scd1* cells), in which case cells were grown first in PMG (i.e. without thiamine) and then in PMG plus 20 µM thiamine for 24 h prior to use in imaging experiments. In all other experiments (i.e. all experiments not involving *scd1^low^* cells) YE5S was used. Supplements such as adenine, leucine and uracil were used at 175 mg/l. Solid media used 2% Bacto agar (Becton Dickinson).

### Plasmid and yeast strain construction

Mating for genetic crosses (Ekwall and Thon, 2017) was performed on SPA5S plates with supplements at 45 mg/l. Crosses were performed using tetrad dissection or random spore analysis. Tagging and deletion of genes were performed using PCR-based methods (Bahler et al., 1998), with the exception of the strains described below, which involved integration of newly constructed plasmids. All plasmid constructions (below) were confirmed by sequencing. For rapamycin-induced dimerization, endogenous Gef1 and Tea1 were tagged with Frb/Frb-GFP (Gef1) and 2FKBP12 (Tea1), using PCR-based methods (Ding et al., 2014). To prevent rapamycin-based inhibition of normal cellular pathways via the endogenous TOR system, these alleles were crossed into a *tor2-S1837E fkh1*Δ background (note that *tor2-S1837E* is different from the *tor1-S1834E* allele described in Ding et al.) (Ding et al., 2014; Laor et al., 2014; Takahara and Maeda, 2012). All strains used in this study are listed in Table S1.

#### *adh13:wee1* plasmid/strain construction

The *wee1* open reading frame (ORF) was amplified by PCR from genomic DNA and cloned into the *Nde*I site of pNATZA13 (kind gift from Y. Watanabe, Francis Crick Institute, London, UK) to form pNATZA13-Wee1 (pKS1448). *Apa*I-linearized pKS1448 was then integrated at the Z locus (Sakuno et al., 2009) of KS515, and positive clones were screened by microscopy and confirmed by colony PCR.

#### *gef1-EANA-3mCherry* plasmid/strain construction

TOPO-Gef1-3mCherry:kan plasmid (pKS1632) was constructed using a three-piece Gibson assembly approach (NEB). Briefly, PCR fragments of TOPO vector (pCR2.1), Gef1 ORF (flanked by 180 bp upstream of Gef1 ORF), and 3mCherry-Kan fragment (flanked by 180 bp downstream of Gef1 ORF) were assembled to generate pKS1632. A PCR fragment of Gef1 (internal fragment corresponding to amino-acid residues 314-508 but containing two point mutations, E318A and N505A) was subsequently introduced into pKS1632 via a two-piece Gibson assembly approach to generate pKS1699. A Gef1-containing *Spe*I-*Xba*I fragment from pKS1699 was then purified and transformed into strain KS7656 to generate strain KS9183.

#### *gef1-3mCherry-GFP* plasmid/strain construction

The *gef1+* ORF was amplified from genomic DNA and introduced into pINTH41.3HA-mCherry-GBP-3PK:natMX6 plasmid (kind gift from I. Hagan, Cancer Research UK Manchester Institute, Manchester, UK) via a two-piece Gibson assembly to generate pKS1488. *Not*I-linearized pKS1488 was then transformed into strain KS7742 to generate strain KS8152.

### Microscopy sample preparation and imaging

All imaging experiments were performed with exponentially growing cells cultured at 25°C. Imaging was performed either in coverslip dishes (MatTek; P35G-0.170-14-C.s) or four-chamber glass bottom microslides (Ibidi; 80427). Imaging dishes/slides were placed on a 25°C heat block, coated with 1 mg/ml soybean lectin (Sigma-Aldrich; L1395), left for 10 min and washed with appropriate medium to remove excess lectin. Log-phase culture was added to dishes/slides and left to settle for 15 min. The dishes/slides were washed extensively with media using aspiration with at least three full exchanges of media (approximately 1 ml each). Finally, 500 µl of medium was added to the dish/slide before imaging.

For lectin-free imaging, a four-chamber microslide was used without any lectin coating in the relevant chamber. Then, 300 µl of *gef1-3mCitrine* culture (OD_595_=0.25) was added directly to that chamber and imaged within 10 min. Cells immobilized on a lectin-coated glass bottom in an adjacent chamber were used to first find the correct focal plane for imaging.

Live-cell fluorescence imaging was performed using a custom spinning-disk confocal microscope unit [Nikon TE2000 microscope base, attached to a modified Yokogawa CSU-10 unit (Visitech) and an iXon+ Du888 EMCCD camera (Andor), 100×/1.45 NA Plan Apo objective (Nikon), Optospin IV filter wheel (Cairn Research), MS-2000 automated stage with CRISP autofocus (ASI), and thermo-regulated chamber maintained at 25°C (OKOlab)]. Metamorph software (Molecular Devices) was used to control the spinning-disk confocal microscope.

The 3-BrB-PP1 (A602985) was obtained from Toronto Research Chemicals and dissolved in methanol to make a 50 mM stock solution. It was used at a final concentration of 8 µM; 4 µM was insufficient to completely prevent mitotic entry. Thiamine, MBC and TBZ were obtained from Sigma-Aldrich. Thiamine was dissolved in water as 200 mM stock and used at a final concentration of 20 µM. MBC stock solution was 2.5 mg/ml in dimethyl sulfoxide (DMSO) and was used at a final concentration of 25 µg/ml (therefore 1% DMSO final concentration). In MBC experiments, for the DMSO-only control, a 1% DMSO final concentration was used. TBZ stock solution was 30 mg/ml in DMSO and was used at a final concentration of 150 µg/ml (therefore 0.5% DMSO final concentration). Rapamycin was obtained from Fisher Scientific (10798668). Rapamycin was dissolved in DMSO as a 1 mg/ml stock and used at a final concentration of 2.5 µg/ml. All drug additions during imaging were performed by medium exchange using a 1 ml polyethylene transfer pipette (Fisher Scientific, 1346-9118).

We note that when grown on solid PMG medium without thiamine, *scd1^low^ tea1*Δ*, scd1^low^ tea4*Δ*, and scd1^low^ pom1*Δ double mutants formed colonies that were noticeably smaller than wild-type cells and *scd1^low^* single mutants. Under these conditions, the double mutants also showed some defects in septum positioning and in completion of cytokinesis. Accordingly, we found that during normal growth in liquid PMG medium without thiamine, 22% (9/41) of nondividing *scd1^low^ tea1*Δ cells were binucleate, compared with 0% (0/87) of *scd1^low^* (*tea1+*) cells. After 9 h repression in thiamine, 58% (32/55) of nondividing *scd1^low^ tea1*Δ cells were binucleate, compared with 0% (0/151) of *scd1^low^* (*tea1+*) cells. In all *scd1^low^* mutants, analysis of growth patterns after inhibition of Cdc2-asM17 by 3-BrB-PP1 was limited to mononucleate cells.

Numbers of independent biological replicate experiments are provided for each yeast strain, in each figure, in the yeast strain list in Table S1. We define an independent biological replicate as growing/culturing a given yeast strain and then using it for a given biochemistry experiment or imaging session. Up to a few hundred cells of the same genotype may be imaged in any given replicate imaging session.

### Analysis of microscopy images

Processing of the acquired raw images was executed using ImageJ (Fiji, NIH). Unless otherwise stated, all images and videos shown are maximum projections of eleven *Z*-sections with 0.7 μm step-size. For rigid body registrations, ImageJ StackReg and Linear Stack Alignment with SIFT plugins were used. Image formatting and assembly were performed using Photoshop (Adobe) and Illustrator CS3 (Adobe). Cell outlines were drawn by hand in Illustrator, using images from individual video timepoints as templates. In some cases, cell outlines were aligned slightly, owing to limited cell movement during imaging. In a few cases where cell borders were more difficult to discern (e.g. in late-stage depolarized cells), images from successive timepoints were superimposed and then used as a template for drawing. Videos were edited using ImageJ and QuickTime (Apple).

Quantification of the percentage of cells with polarized mCherry-Bgs4 or GFP-Bgs4 signals on cell tips (Figs 2, 3, 4 and 6) was performed manually, based on analysis of videos. Cells with persistent mCherry-Bgs4 or GFP-Bgs4 signals on the cell tips (over a period of 4 h) were scored as polarized cells. To avoid confusing depolarized cells with cells that simply had a diminished Bgs4 signal (because of photobleaching), quantification was performed using only on the first 4 h of videos. Occasional cells that transiently lost the tip signal but then regained it shortly afterwards (i.e. in the same place) were also scored as polarized cells. Graphs were created using GraphPad Prism software. Statistical analysis was carried out using online tools (http://www.graphpad.com/quickcalcs/; http://www.socscistatistics.com).

## Supplementary Material

Supplementary information
