# Supplementary material for: Local and global Cdc42 guanine nucleotide exchange factors for fission yeast cell polarity are coordinated by microtubules and the Tea1–Tea4–Pom1 axis
Source: J Cell Sci. 2018 Jul 19;131(14):jcs216580. doi: 10.1242/jcs.216580 (PMC6080602; doi:10.1242/jcs.216580)
Supplement: Supplementary information [file joces-131-216580-s1.pdf]

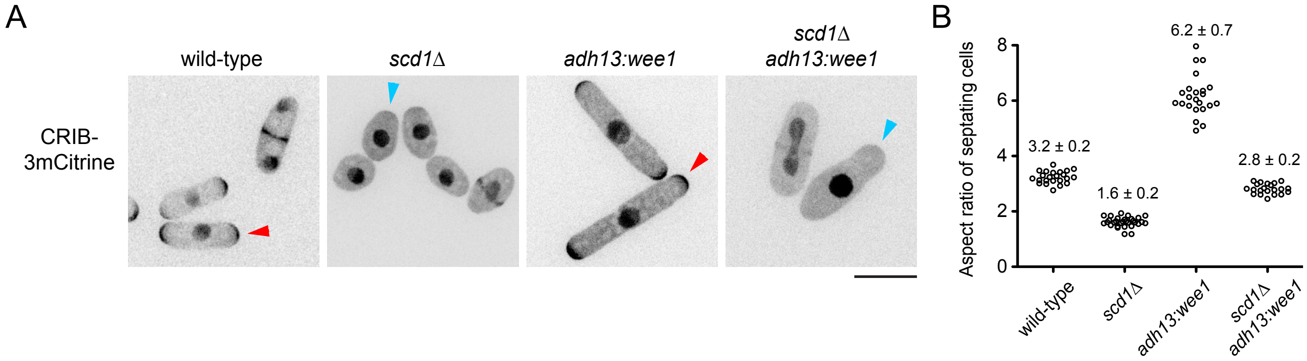


**Figure S1. Overexpression of mitotic inhibitory kinase Wee1 reveals polarized growth of *scd1*Δ cells.** **(A)** Cell morphology and distribution of Cdc42-GTP reporter CRIB-3mCitrine in cells of the indicated genotypes. Polarized shape of *scd1*Δ cells is seen upon *wee1* overexpression. Arrowheads indicate detection (red) or no significant detection (blue) of CRIB at cell tips. **(B)** Aspect ratio (cell length divided by cell width) of septating cells of the indicated genotypes, with mean and SD. Wild-type and *scd1*Δ *adh13:wee1* cells have similar ratios. Numbers of cells scored were: 23 (wild-type), 30 (*scd1*Δ*)*, 23 (*adh13:wee1*), and 21 (*scd1*Δ *adh13:wee1*). Bar, 10 μm.

Journal of Cell Science • Supplementary information

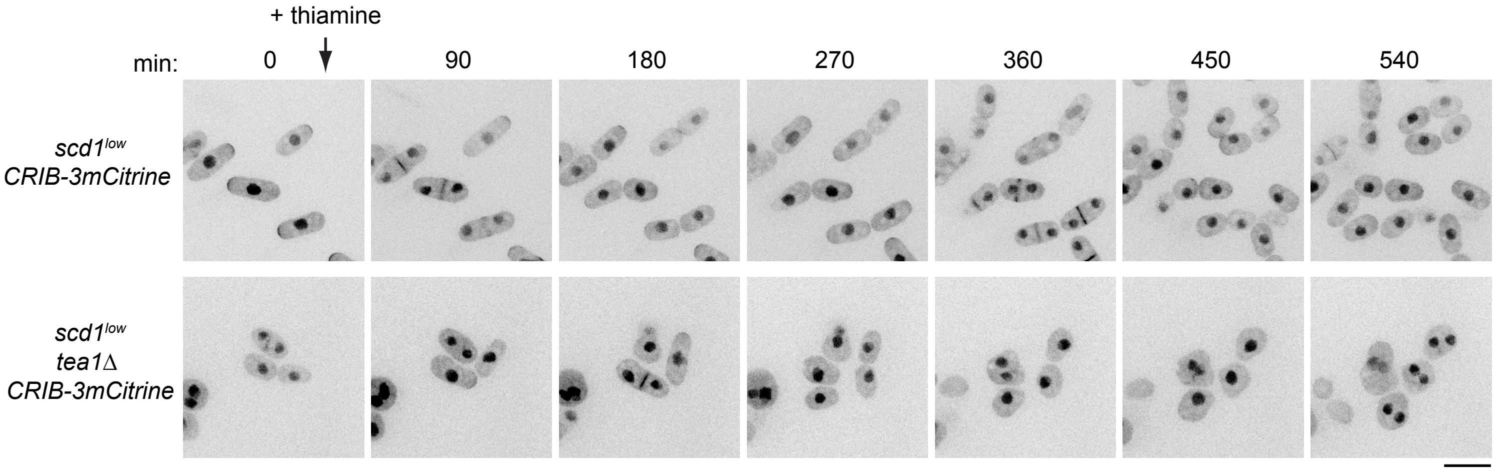


**Figure S2. Growth of *scd1*$^{low}$ and *scd1*$^{low}$ *tea1*Δ cells expressing wild-type Cdc2.**
Cell morphology and CRIB-3mCitrine localization in the indicated genotypes. These cells also express Lifeact-mCherry (not shown). Thiamine was added to repress *nmt81:3HA-scd1* expression just after the 0 min time-point; therefore at early time-points, cells have relatively higher levels of Scd1 and thus more detectable CRIB at cell tips (see also Fig. 3A). Effects of *scd1* repression are apparent from 270 min onwards. Note increased wide/round cell shape in *scd1*$^{low}$ cells over time (top panels), and isotropic-like growth and extremely round shape in *scd1*$^{low}$ *tea1*Δ cells (bottom panels). Binucleate cells in *scd1*$^{low}$ *tea1*Δ are likely related to defects in cytokinesis caused by round shape. Some *scd1*$^{low}$ *tea1*Δ cells are binucleate even before repression, but not all *scd1*$^{low}$ *tea1* Δ cells become binucleate after repression (see also Fig. S3A and Methods). Bar, 10 µm. See also Movie 3.

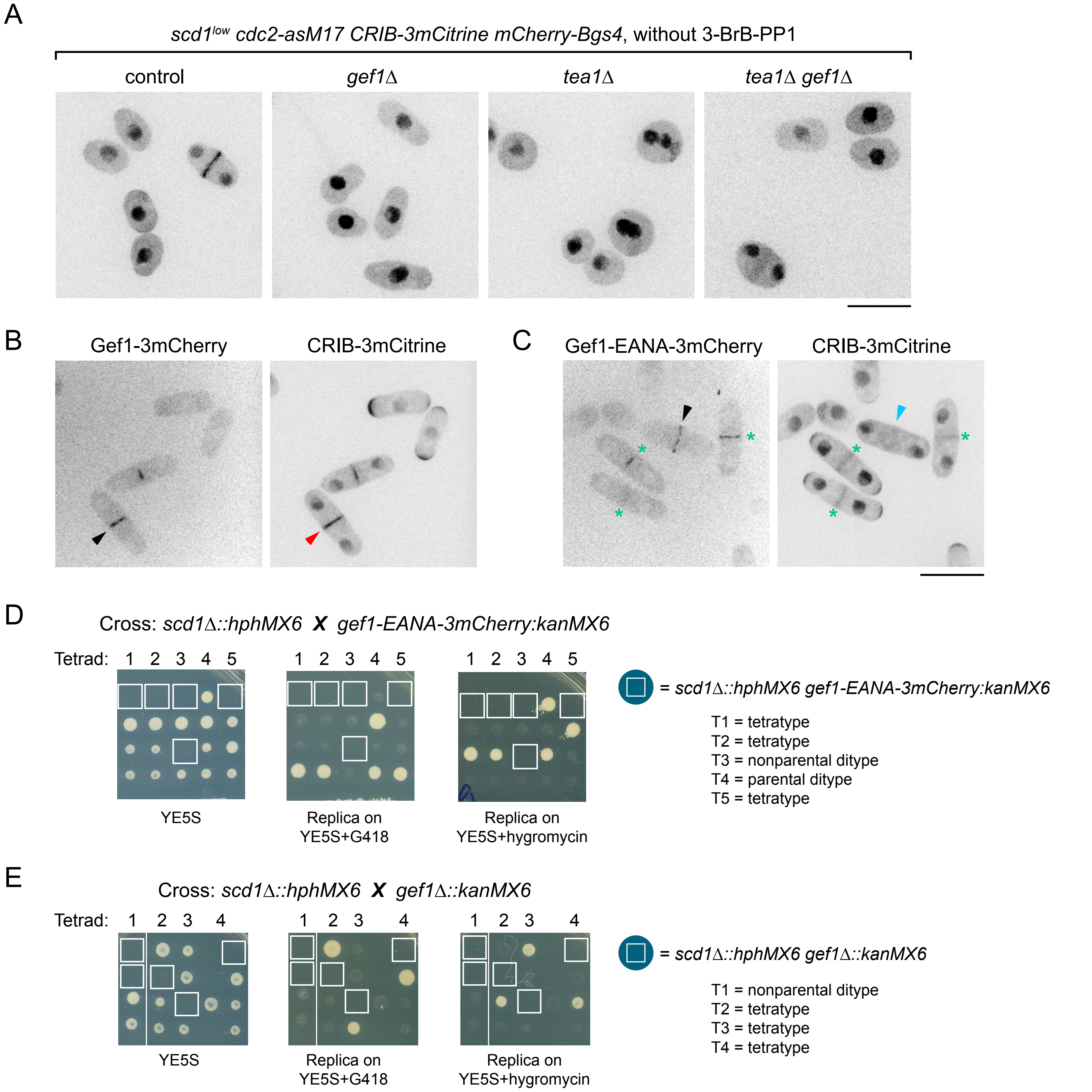


**Figure S3. Supporting data for restoration of polarized growth in *scd1*$^{low}$ *tea1*Δ cells after further loss of *gef1* function**

**(A)** Cell morphology in the indicated genotypes during exponential growth, after 24 hr *scd1* repression. 3-BrB-PP1 was not added to cultures, and thus *cdc2-asM17* was not inhibited. CRIB-3mCitrine signal is shown here as a marker for cell volume and cell nuclei. Note that *tea1*Δ cells are round and often binucleate (see Methods), while *tea1*Δ *gef1*Δ cells are more similar to *gef1*Δ and control cells. **(B,C)** *Gef1-EANA-mCherry* is a loss-of-function mutation. In septating cells, both wild-type Gef1-3mCherry (B) and mutant Gef1-EANA-3mCherry (C) localize to the division site (black arrowheads). Wild-type Gef1-3mCherry promotes Cdc42-GTP (CRIB-3mCitrine) accumulation at the division site during early stages of septation (red arrowhead), but Gef1-EANA-3mCherry does not (blue arrowhead). In later stages of septation, CRIB-3mCitrine at the division site is more diffuse and weak and does not correlate with Gef1-EANA-3mCherry (green asterisks); this later localization is known to be independent of Gef1 (Wei et al., 2016). **(D, E)** Tetrad analyses showing synthetic lethality of *gef1-EANA-3mCherry* with *scd1*Δ (D) and confirming synthetic lethality of *gef1*Δ with *scd1*Δ (E) (Coll et al., 2003). Spores were germinated on YE5S and replica-plated as indicated. Boxes indicate inferred position of non-viable double mutants. Bars, 10 µm.

Journal of Cell Science • Supplementary information

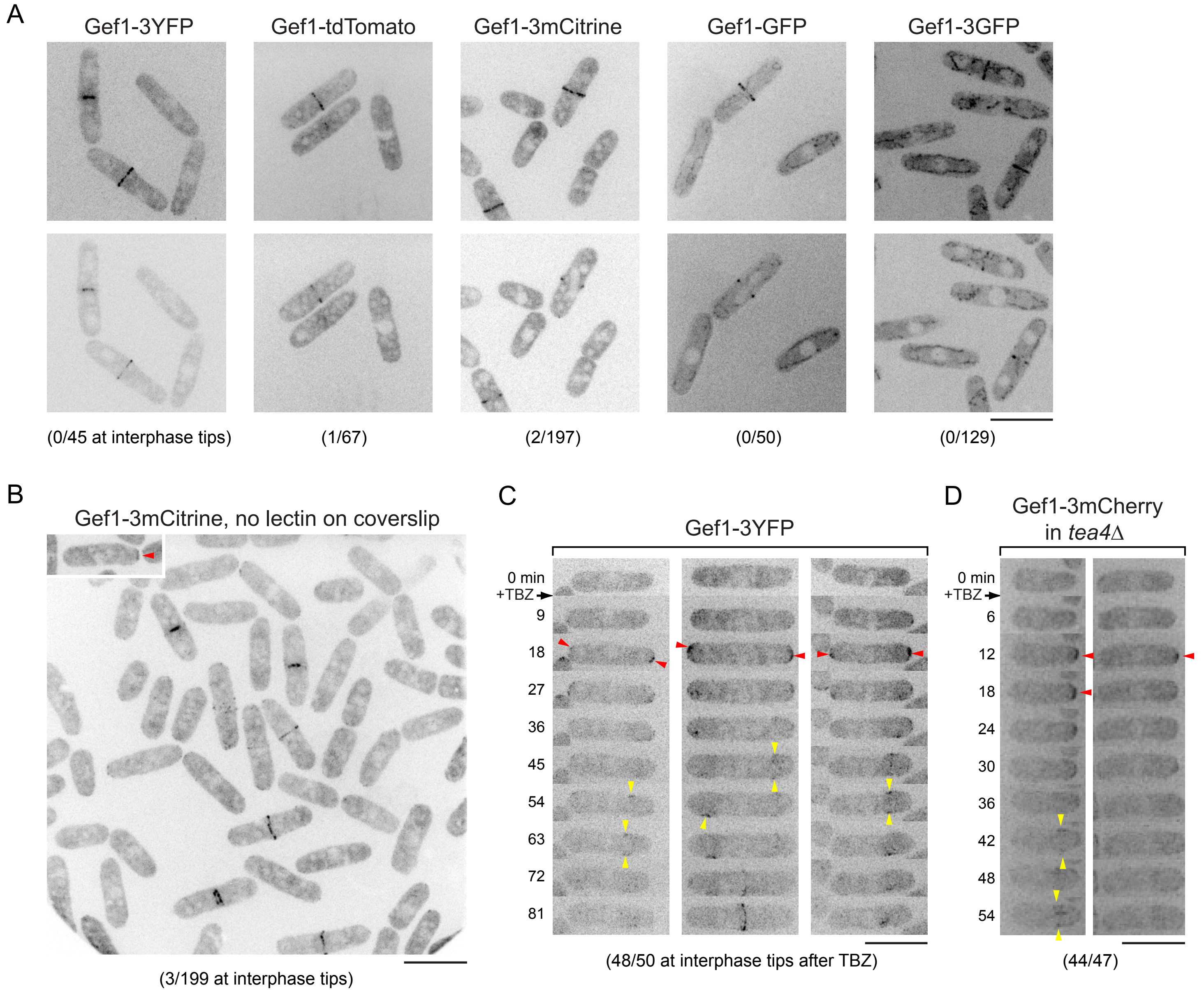


**Figure S4. Gef1 is cytosolic during interphase but transiently localizes to cell tips after TBZ treatment.** **(A)** Still images of Gef1 fused to different fluorescent proteins. Top panels show maximum projections, and bottom panels show corresponding central Z-section or two adjacent central Z-sections. During interphase, Gef1 is cytosolic, and during cell division, Gef1 localizes to the division site. In all cases, cells were grown in YE5S to mid-log phase and imaged under conditions that minimize stress (Mutavchiev et al., 2016; see Methods). In some cases, high exposures were used to confirm absence of Gef1 from cell tips; as a result, mitochondrial autofluorescence is apparent in images of Gef1-GFP and Gef1-3GFP. Numbers of cells with detectable Gef1 at interphase tips are shown below the representative images. **(B)** Gef1-3mCitrine localization in cells imaged within 10 min after adding to uncoated glass-coverslip dishes. Under these conditions, Gef1 is also cytosolic during interphase. This demonstrates that pretreatment of coverslips with soybean lectin (normally used for longer-term imaging; see Methods) does not alter Gef1 localization. Inset shows one interphase cell with Gef1 at cell tips (arrowhead). **(C)** Recruitment of Gef1-3YFP from the cytosol to cell tips after treatment with the microtubule depolymerizing drug thiabendazole (TBZ; 150 μg/ml). TBZ has off-target effects that lead to cell depolarization independently of disrupting microtubules (Sawin and Snaith, 2004). TBZ was added just after imaging the 0 min time-point. After TBZ treatment, Gef1-3YFP transiently localizes to cell tips (red arrowheads) and later localizes more weakly to patches on cell sides (yellow arrowheads), which move towards cell middle. **(D)** Recruitment of Gef1-3mCherry from the cytosol to cell tips after TBZ treatment in *tea4*Δ cells. This demonstrates that Tea4 is not required for TBZ-induced Gef1 cell-tip localization. In (C) and (D), numbers below images indicate number of cells with Gef1 at interphase cell tips within 20 min after TBZ treatment. Bars, 10 μm. See also Movie 5.

Journal of Cell Science • Supplementary information

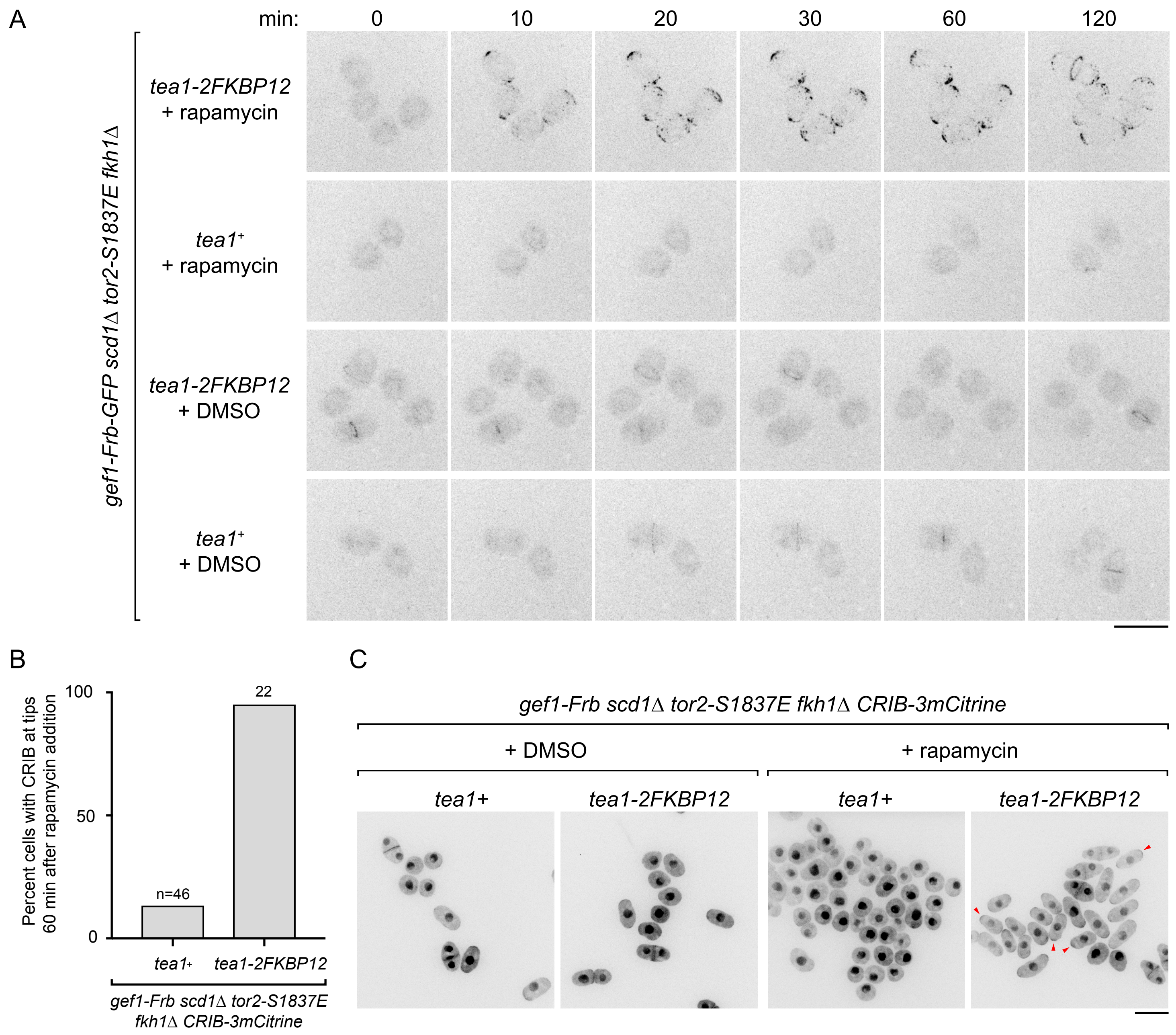


**Figure S5. Targeting Gef1 to cell tips via rapamycin-induced dimerization with Tea1.**
**(A)** Movie time-points showing Gef1-Frb-GFP localization and cell morphology in the indicated genotypes after rapamycin or control DMSO treatment. Gef1-Frb-GFP recruitment to cell tips requires both rapamycin and Tea1-2FKBP12 and leads to increased polarized cell shape. **(B)** Quantification of CRIB-3mCitrine localization at cell tips in the indicated genotypes 60 min after rapamycin addition to target Gef1 to cell tips, from movies of the type shown in Fig. 5D. A small percentage of *tea1*+ cells have detectable CRIB at cell tips, but this is much lower than in *tea1-2FKBP12* cells (see also panels in (C)). Differences were highly significant ($p<0.0001$; Fisher's exact test). **(C)** Cell morphology and CRIB-3mCitrine localization in the indicated genotypes after 16 hr treatment with rapamycin or DMSO. Note polarized cell shape and CRIB localization to cell tips in rapamycin-treated *tea1-2FKBP12* cells (examples indicated by arrowheads). Bars, 10 μm. See also Movie 6.

Journal of Cell Science • Supplementary information

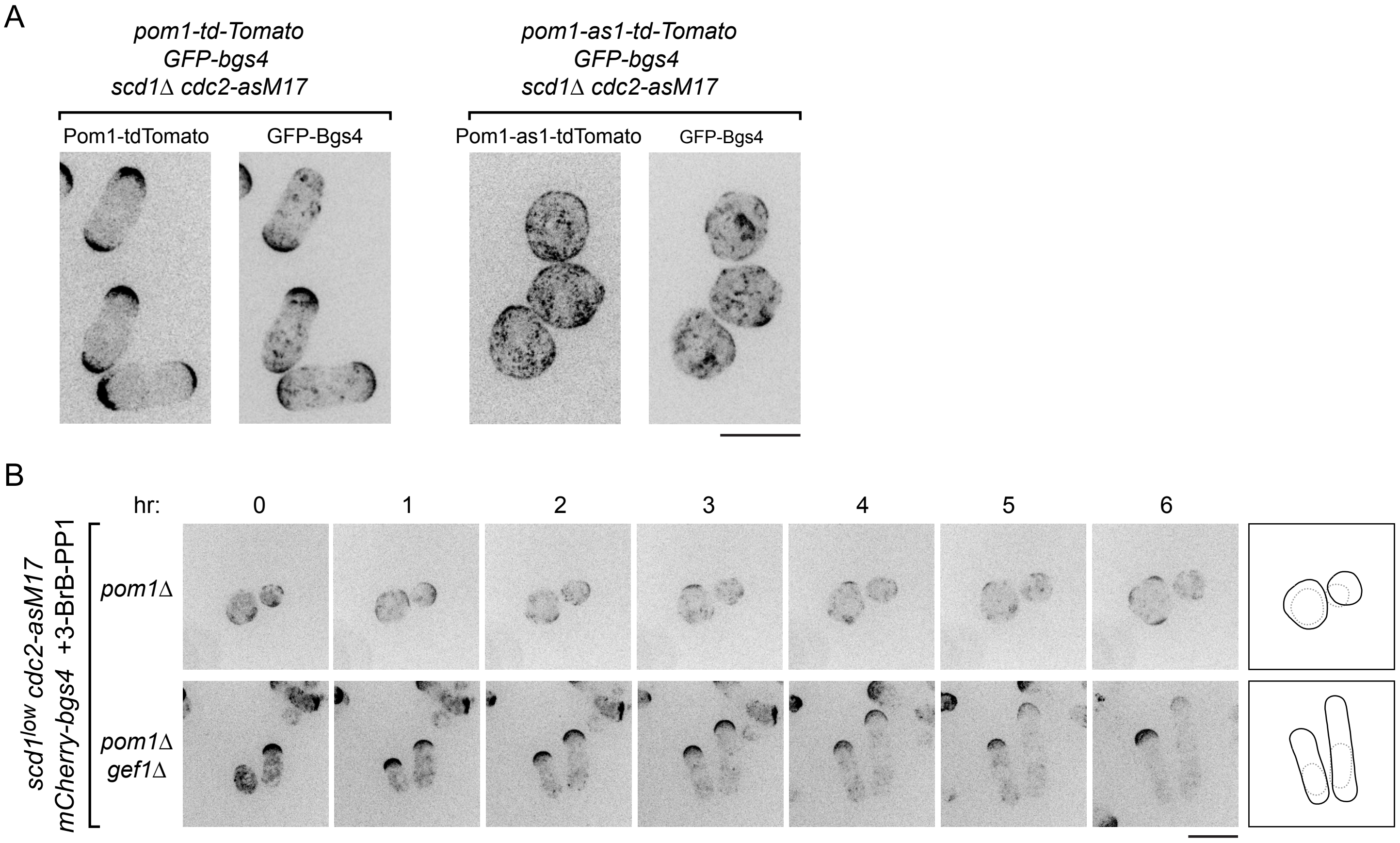


**Figure S6. Supporting data for the role of Pom1 in PORTLI growth.**
**(A)** Single time-point images (i.e. not from movies) of Pom1-tdTomato and GFP-Bgs4, and Pom1-as1-tdTomato and GFP-Bgs4, in the indicated genotypes after 4 hr 3-BrB-PP1 treatment. Experiment was as in Figure 6, but to avoid photobleaching, no images were acquired prior to those shown here. This demonstrates that membrane-associated Pom1-as-tdTomato is much more homogeneously distributed after inhibition by 3-BrB-PP1 in these cells and that loss of signal from cell tips is not simply due to photobleaching. **(B)** Deletion of *gef1* restores polarized growth to *scd1*$^{low}$ *pom1*Δ cells. Movie time-points showing cell morphology and mCherry-Bgs4 distribution in indicated genotypes. *scd1* expression was repressed 24 hr before imaging. 3-BrB-PP1 was added 30 min before imaging. Diagrams show cell outlines at beginning and end of movies; outlines were aligned slightly to account for limited cell movement. Bars, 10 µm. See also Movie 8.

Journal of Cell Science • Supplementary information

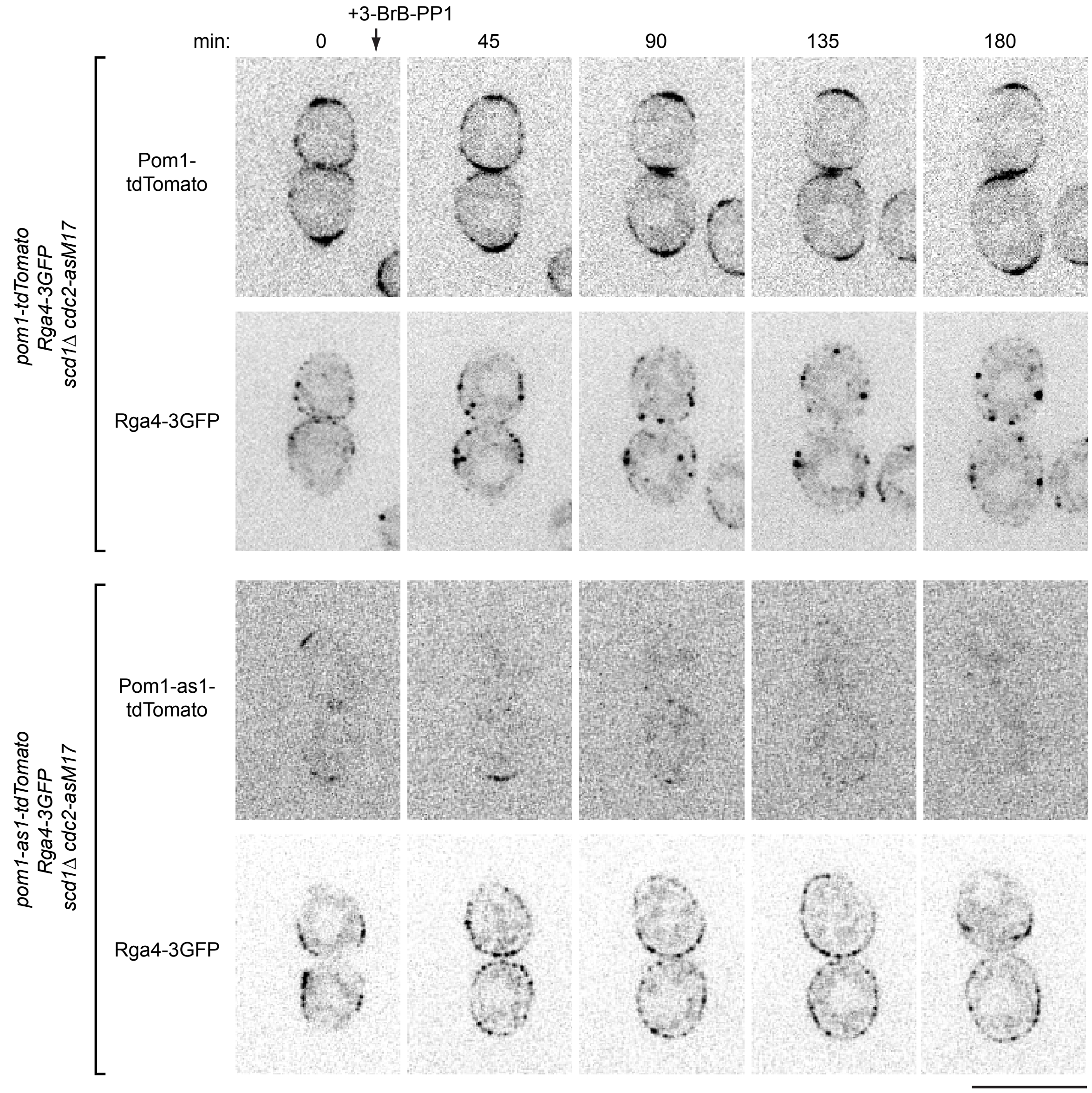


**Figure S7. Single-channel images of Pom1-tdTomato, Pom1-as1-tdTomato and Rga4-3GFP after 3-BrB-PP1 addition.**
Single-channel images corresponding to the merged images shown in Figure 6C. Note that at some time-points, some Rga4-GFP signal appears to internal (i.e. not on the plasma membrane). Bars, 10 µm.

Journal of Cell Science • Supplementary information

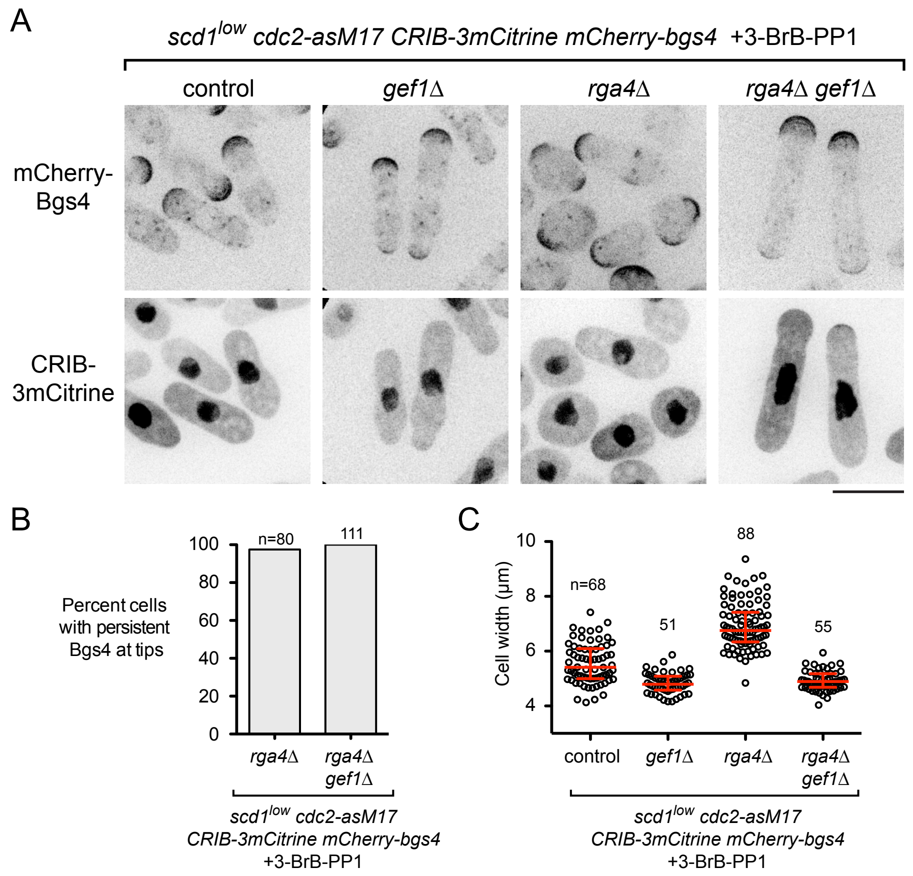


**Figure S8. Polarity defects in *rga4*Δ *scd1*$^{low}$ cells during extended interphase are rescued by *gef1*Δ.**

**(A)** Cell morphology, mCherry Bgs4-localization and CRIB-3mCitrine distribution in the indicated genotypes after 3-BrB-PP1 treatment. *scd1* expression was repressed for 24 hr before addition of 3-BrB-PP1. Cells were imaged 5 hr after addition of 3-BrB-PP1. CRIB-3mCitrine signal shows cell dimensions and was used to measure cell width in C. Note that *rga4*Δ cells in *scd1*$^{low}$ background are wider/rounder than other genotypes, although polarity defects are not as strong as in *scd1*Δ background (see Figure 7A). **(B)** Quantification of mCherry-Bgs4 at cell tips in the indicated genotypes, from experiments in A. **(C)** Cell width for the indicated genotypes from images as in A, 5 hr after addition of 3-BrB-PP1. Median and interquartile ranges are shown. All pairwise differences were highly significant ($p$<0.0001; Mann-Whitney test), except *gef1*Δ vs. *rga4*Δ *gef1*Δ ($p$=0.20). n indicates number of cells scored. Bar, 10 µm.

Journal of Cell Science • Supplementary information

## MOVIES

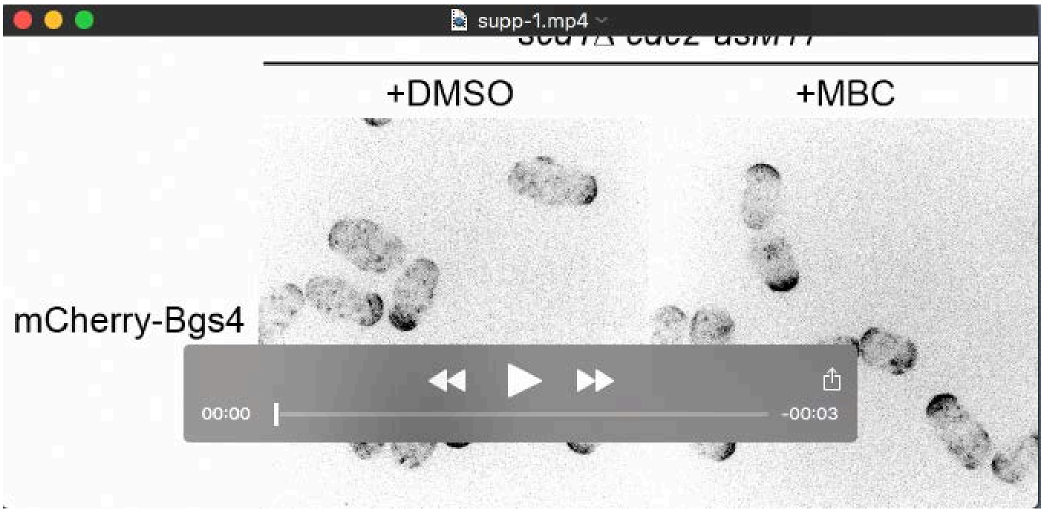


**Movie 1. Microtubule depolymerization in *scd1*Δ cells leads to PORTLI growth.** mCherry-Bgs4 distribution and cell morphology of *scd1*Δ *cdc2-asM17 mCherry-bgs4* cells. Bgs4 on the plasma membrane indicates sites of growth. Cells were pretreated with 3-BrB-PP1 60 min prior to start of imaging, to inhibit Cdc2 kinase activity, and then treated with either DMSO or MBC at start of imaging (still in presence of 3-BrB-PP1). For DMSO treatment, cell at lower right corresponds to cell shown in Fig. 2A. For MBC treatment, cell at mid-lower center corresponds to cell shown in Fig. 2A. Time interval during acquisition, 10 min; total elapsed time, 420 min; time compression at 15 frames per second playback, 9000X.

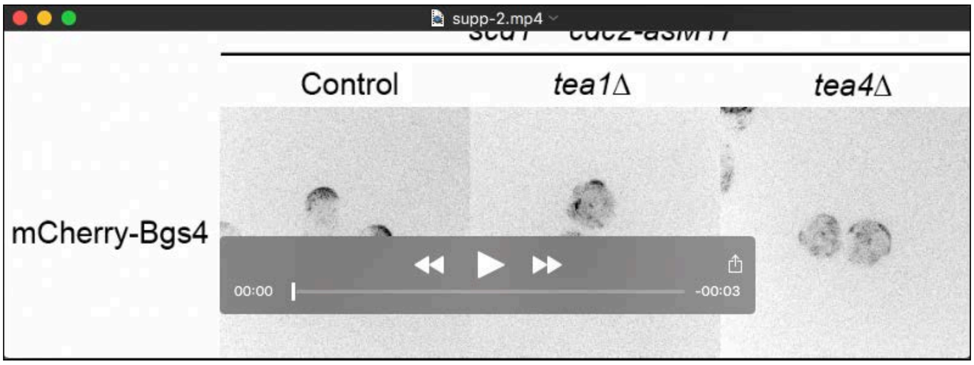


**Movie 2. When *scd1* is expressed at very low levels, *tea1*Δ and *tea4*Δ cells show PORTLI growth.** mCherry-Bgs4 distribution and cell morphology in control cells, *tea1*Δ, and *tea4*Δ cells, all in a *scd1$^{low}$ cdc2-asM17 mCherry-bgs4* genetic background. Bgs4 on the plasma membrane indicates sites of growth. Cells correspond to those shown in Fig. 3B. *scd1* expression was repressed by thiamine addition 24 hr prior to start of imaging. Cdc2 kinase activity was inhibited by 3-BrB-PP1 addition 30 min before imaging. Time interval during acquisition, 10 min; total elapsed time, 420 min; time compression at 15 frames per second playback, 9000X.

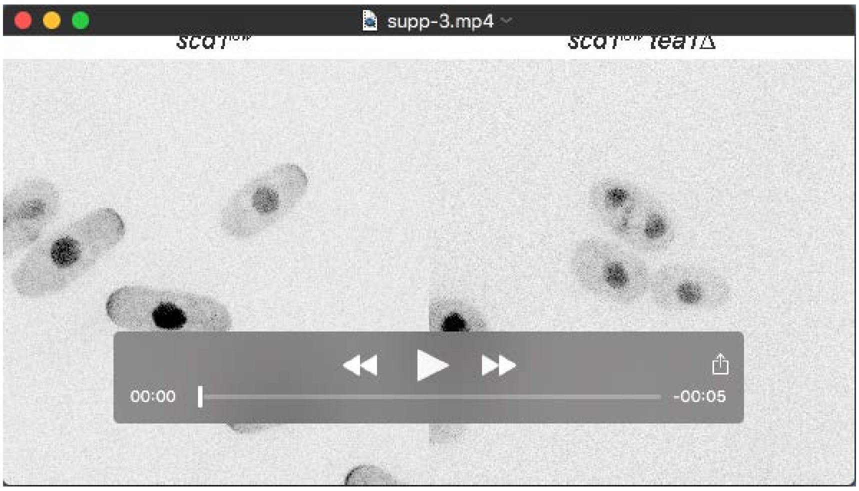


**Movie 3. Growth of *scd1*$^{low}$ and *scd1*$^{low}$ *tea1*Δ cells expressing wild-type Cdc2.** CRIB-3mCitrine distribution and cell morphology in exponentially-growing *scd1*$^{low}$ and *scd1*$^{low}$ *tea1*Δ cells expressing wild-type Cdc2. Thiamine was added to repress *scd1* expression just after the first time-point, which is paused in the movie. Cells correspond to those shown in Fig. S2. Effects of *scd1* repression, including isotropic-like growth of *scd1*$^{low}$ *tea1*Δ cells, become apparent about half-way through the movie. See Fig. S2 legends and Methods for further details. Time interval during acquisition, 9 min; total elapsed time, 540 min; time compression at 15 frames per second playback, 8100X.

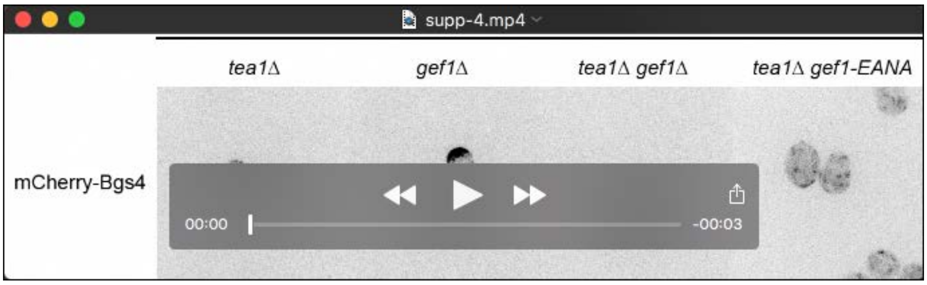


**Movie 4. Loss of *gef1* function restores polarized growth to *scd1*$^{low}$ *tea1*Δ cells.** mCherry-Bgs4 distribution and cell morphology in *tea1*Δ, *gef1*Δ, *tea1*Δ *gef1*Δ and *tea1*Δ *gef1-EANA* cells, all in a *scd1*$^{low}$ *cdc2-asM17 mCherry-bgs4* genetic background. Bgs4 on the plasma membrane indicates sites of growth. Cells correspond to those shown in Fig. 4A. *scd1* expression was repressed by thiamine addition 24 hr prior to start of imaging. Cdc2 kinase activity was inhibited by 3-BrB-PP1 addition 30 min before imaging. A transient loss of Bgs4 from cell tips is seen in some *scd1*$^{low}$ *gef1* mutant cells (~20%), including some of the examples shown in the movie; the reasons for this are not completely clear. Time interval during acquisition, 10 min; total elapsed time, 420 min; time compression at 15 frames per second playback, 9000X.

Journal of Cell Science • Supplementary information

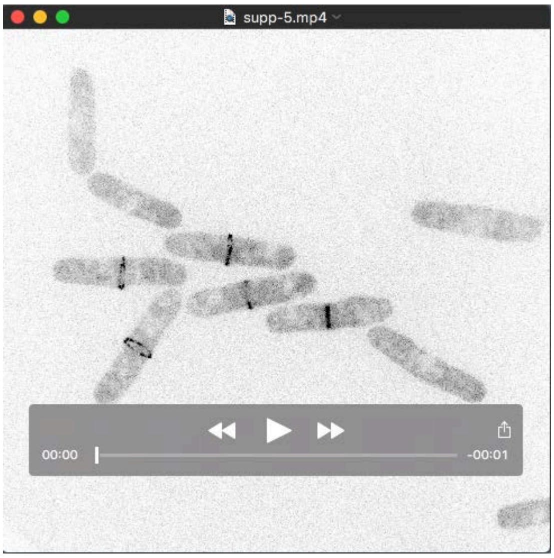


**Movie 5. Gef1-3YFP is transiently recruited to the cell tips upon TBZ treatment.** TBZ was added just after the first time-point, which is paused in the movie. Prior to TBZ addition, Gef1-3YFP in dividing cells is present at the division site and in the cytoplasm, and Gef1-3YFP in interphase cells is uniformly distributed in the cytoplasm, without any visible enrichment at the cell tips. Upon TBZ addition, interphase Gef1-3YFP signal is transiently observed at cell tips and later appears to move along the cell cortex towards the cell middle. Three of the cells in the movie correspond to those shown in Fig. S4C. Time interval during acquisition, 9 min; total elapsed time, 81 min; time compression at 15 frames per second playback, 8100X.

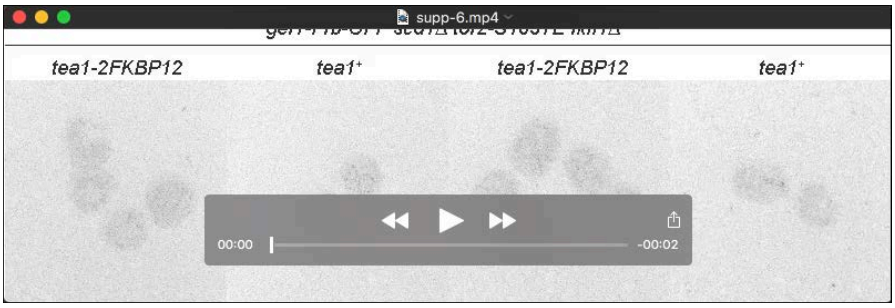


**Movie 6. Rapamycin addition to *gef1-Frb-GFP tea1-2FKBP12* cells leads to recruitment of Gef1-Frb-GFP to cell tips and increased cell polarization.** Gef1-Frb-GFP localization and cell morphology in *tea1-2FKBP12* cells and in control cells expressing untagged Tea1 (*tea1+*), all in *scd1Δ tor2-S1837E fkh1Δ* genetic background, after addition of rapamycin or control DMSO. Cells correspond to those shown in Fig. S5A. Rapamycin or DMSO were added just after the sixth time point. Time interval during acquisition, 5 min; total elapsed time, 150 min; time compression at 15 frames per second playback, 4500X

Journal of Cell Science • Supplementary information

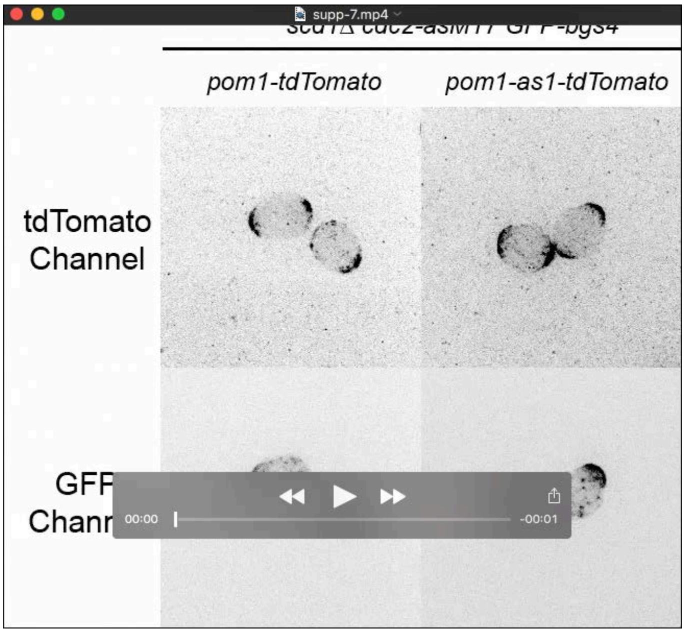


**Movie 7. Inhibition of Pom1 kinase activity in *scd1*Δ cells leads to PORTLI growth.** Cell morphology and distribution of Pom1-tdTomato and GFP-Bgs4, or Pom1-as1-tdTomato and GFP-Bgs4, in *scd1*Δ *cdc2-asM17* genetic background after 3-BrB-PP1 treatment. Bgs4 on the plasma membrane indicates sites of growth. Cells correspond to those shown in Fig. 6A. 3-BrB-PP1 inhibits activity of both Cdc2-asM17 and Pom1-as1-tdTomato and was added just after the first time-point. Note that 3-BrB-PP1 treatment depolarizes Pom1-as1-tdTomato, and this leads to PORTLI growth. Time interval during acquisition, 20 min; total elapsed time, 240 min; time compression at 15 frames per second playback, 18,000X.

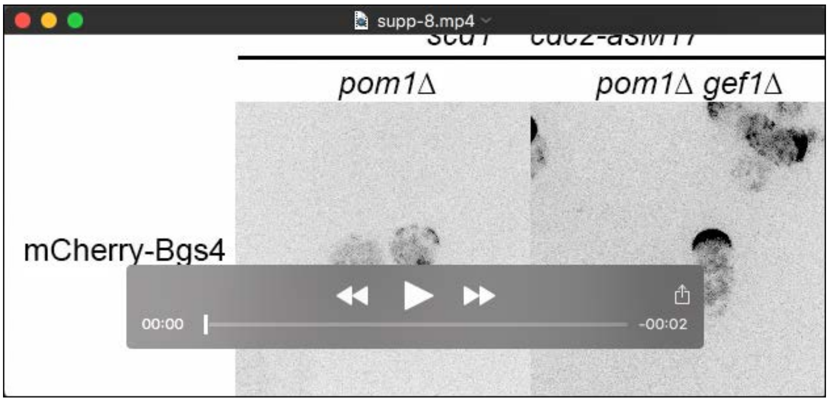


**Movie 8. Deletion of *gef1* restores polarized growth to *scd1$^{low}$ pom1*Δ cells.** mCherry-Bgs4 distribution and cell morphology of *pom1*Δ and *pom*Δ *gef1*Δ mutants in *scd1$^{low}$ cdc2-asM17 mCherry-bgs4* genetic background. Bgs4 on the plasma membrane indicates sites of growth. Cells correspond to those shown in Fig. S6B. *scd1* expression was repressed by thiamine addition 24 hr prior to start of imaging. Cdc2 kinase activity was inhibited by 3-BrB-PP1 addition 30 min before imaging. Time interval during acquisition, 10 min; total elapsed time, 350 min; time compression at 15 frames per second playback, 9000X.

Journal of Cell Science • Supplementary information

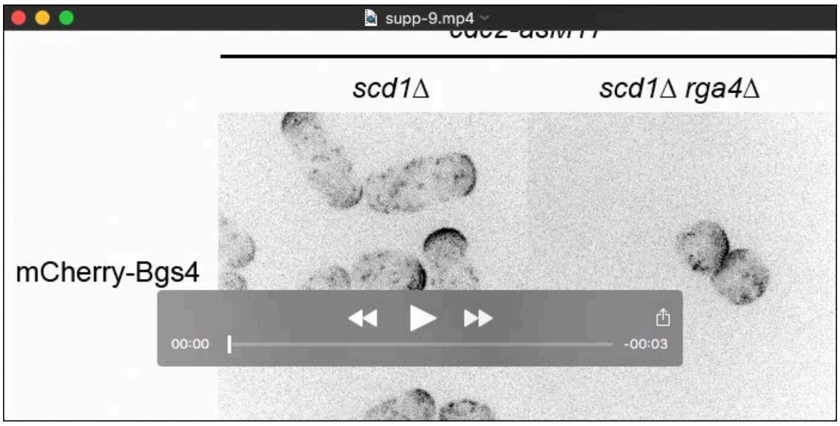


**Movie 9. Deletion of *rga4* leads to PORTLI growth in *scd1*Δ cells.** mCherry-Bgs4 distribution and cell morphology of *scd1*Δ and *scd1*Δ *rga4*Δ mutants in *cdc2-asM17 mCherry-bgs4* background. Bgs4 on the plasma membrane indicates sites of growth. Cells correspond to those shown in Fig. 7A, with slightly larger fields. Cdc2 kinase activity was inhibited by 3-BrB-PP1 addition 30 min before imaging. Time interval during acquisition, 12 min; total elapsed time, 480 min; time compression at 15 frames per second playback, 10,800X.

**Table S1: Yeast strains used in this work, listed by figure**

Click here to Download Table S1
